# Conversion of tectonic and climatic forcings into records of sediment supply and provenance

**DOI:** 10.1038/s41598-019-39754-6

**Published:** 2019-03-11

**Authors:** Glenn R. Sharman, Zoltan Sylvester, Jacob A. Covault

**Affiliations:** 1 Department of Geosciences, University of Arkansas, Fayetteville, AR 72701 USA; 2 Bureau of Economic Geology, Jackson School of Geosciences, The University of Texas at Austin, Austin, TX 78758 USA

## Abstract

Understanding how environmental forcings (e.g., tectonics, climate) are transformed by erosional landscapes into sedimentary signals is a critical component of inverting the stratigraphic record. Previous research has largely focused on sediment supply (*Q*_*s*_) and grain size as the de facto sedimentary signals of changing forcing mechanisms. We use a numerical model to consider the paired response of sediment provenance (*P*_*v*_), expressed as fractional sediment load, and *Q*_*s*_ to demonstrate that the same change in environmental forcing may have a different expression in the sedimentary record. While *Q*_*s*_ reflects integrated denudation across an erosional catchment, *P*_*v*_ is controlled by spatially variable erosion that occurs in transient landscapes. *P*_*v*_ from proximal sediment sources increases during upstream knickpoint migration, whereas *P*_*v*_ from distal sediment sources increases when bedrock channels incise to produce lower gradient profiles. Differences between the *Q*_*s*_ and *P*_*v*_ signals relate to distinct geomorphic processes that operate on different time scales and allow for a refined differentiation of the timing and mechanism of forcings than possible via analysis of either signal alone. Future efforts to integrate multiple sedimentary signals may thus yield a richer picture of underlying forcing mechanisms, facilitating efforts to invert the stratigraphic record.

## Introduction

Sedimentary deposits represent an important archive of how environmental conditions (e.g., tectonics, climate) have changed over geologic time. Using the stratigraphic record to differentiate and reconstruct such environmental forcing mechanisms is of critical importance, as understanding how the Earth’s surface responded to past change is key to predicting the response of the Earth to ongoing and future change, such as a warming climate^1^. A large body of research, based in part on analog and numerical experiments, has highlighted a number of challenges in using sedimentary data to reconstruct external forcing mechanisms, including the tendency for sedimentary signals to be non-unique, modified when the forcing timescale is less than the landscape response time, and shredded or attenuated during sediment transport and deposition^2–8^. Others have found success in inverting the stratigraphic record, but under a limited set of scenarios (e.g., down-system grain size trends^9–12^).

Previous research has largely considered sediment supply (*Q*_*s*_), and to a lesser extent grain size, as the de facto sedimentary signal of changing forcing mechanisms^4,5,11–16^. Although *Qs* is an important sedimentary parameter (e.g., influences system progradation versus retrogradation) and is readily measured in numerical^4,15,16^ and analog^17^ experiments, *Q*_*s*_ is notoriously difficult to constrain in modern, and particularly ancient, sedimentary systems^12,18^. Sedimentary provenance (*P*_*v*_) can be interpreted in both modern and ancient systems via numerous, different proxies that are applicable to gravel, sand, and mud grain size fractions^19,20^. We define *P*_*v*_ as the proportion of sediment mass eroded from the *i*^th^ source rock type:

1 $${P}_{{v}_{i}}( \% )={c}_{i}{Q}_{{s}_{i}}/\sum _{j=1}^{n}{c}_{j}{Q}_{{s}_{j}}$$

where *c* is a dimensionless coefficient that reflects the abundance of a given provenance indicator in a source area and *n* is the number of sediment sources within the catchment area^21^. Other sedimentary data types (e.g., sediment composition, thermochronology, stratigraphic architecture) may also signal changing forcing mechanisms, and how such signals are propagated and preserved in sedimentary systems is an active research topic^5^.

This study explores the paired response of *Q*_*s*_ and *P*_*v*_ to temporally variable tectonic and climatic forcings on an erosional landscape. Specifically, we aim to answer the following questions. (1) Do *Q*_*s*_ and *P*_*v*_ differ in response to stepped and periodic changes in uplift and precipitation rates? (2) What are the underlying geomorphic causes for differences between *Q*_*s*_ and *P*_*v*_? (3) Can the paired *Q*_*s*_ − *P*_*v*_ signal improve the reconstruction of environmental forcing type (tectonics versus climate) and timing?

## Experimental Design and Model Parameters

Following the experimental design of previous workers^16^, we simulate erosion of a two-dimensional landscape using a detachment-limited bedrock channel erosion rate (*E*) of

2 $$E=K{(rA)}^{m}{S}^{n}$$

where *K* is a coefficient of erosion, *r* is precipitation rate, *A* is the upstream drainage area, *S* is the slope, and *m* and *n* are constants that reflect non-linearity between *E*, *rA*, and *S*^22^. Together, the product of *r* and *A* is a proxy for water discharge that scales with precipitation for a catchment of a given size. Although this stream-power incision model is oversimplified with respect to natural rivers^23^, it has been successfully applied to mountainous rivers in different climate regimes^24^ and provides a minimum estimate of system response time from which to evaluate signal transmission from changing forcing mechanisms^25^. Use of the detachment-limited stream-power incision model thus provides an optimistic (best-case) view of signal transmission, as signals are more readily transmitted from landscapes with short, or ‘rapid,’ equilibrium response times, defined as the time elapsed between a forcing perturbation and subsequent achievement of topographic and denudational steady-state^5,13,14,25^. To carry out the landscape evolution modeling, we have used the open-source, Python-based Landlab toolbox^26^, with Equation 2 implemented using the FastscapeEroder algorithm^27^.

**Figure 1:**
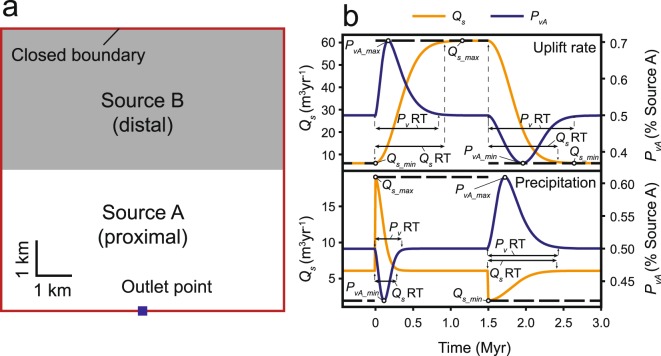
(**a**) Landscape model geometry showing the aerial distribution of Source A and Source B. Variations on this model geometry were used in Scenarios 1.10–1.15 and 2.10–2.15 (Table DR1). (**b**) Explanation of experimental design and terms for a stepped increase and decrease in forcing (thick, dashed black line). X-axis displays elapsed time since initiation of experiment following establishment of steady-state. Scenarios 1.1 (uplift rate) and 2.1 (precipitation) are shown (see Tables DR1 and DR2). Minimum and maximum *Q*_*s*_ and *P*_*vA*_ values attained during stepped forcing are identified as *Q*_*s_min*_, *P*_*vA_min*_, *Q*_*s_max*_, and *P*_*vA_max*_, respectively. Abbreviations: RT-response time.

We follow model parameters of previous workers^16^ with a square grid with dimensions of 64 km^2^ (Fig. 1; model parameters are presented in Table DR1). All four of the model’s bounding edges are closed boundaries with the exception of a single outlet point at the midpoint of the southern boundary that has a fixed value of 0 m elevation (Fig. 1). The model is further divided into two regions (Source A and Source B) positioned proximally and distally to the outlet point, respectively (Fig. 1). These regions represent different rock types in the landscape model for the purpose of modeling change in *P*_*v*_ during landscape evolution.

**Figure 2:**
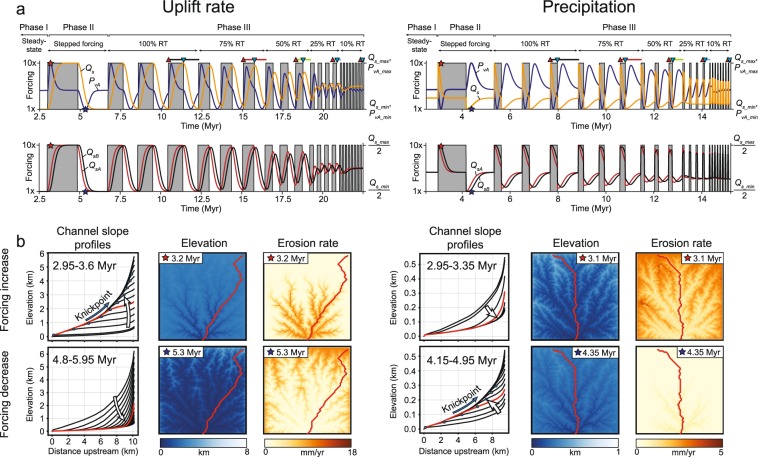
Experimental results. (**a**) Forcing (uplift rate and precipitation) and the corresponding sedimentary signal. Top plot shows *Q*_*s*_ and *P*_*vA*_ (% Source A) and the bottom plot shows the *Q*_*s*_ from Source A and Source B (Q_*sA*_ and *Q*_*sB*_, respectively). See Fig. 1 and text for an explanation. (**b**) Channel slope profiles, map of erosion rate, and elevation during periods of transient landscape response to increased (above) and decreased (below) forcing. Left and right panels correspond to changes in uplift rate and precipitation, respectively, shown in (**a**). The maps correspond to the channel slope profile colored red and to the red and blue stars in (**a**). Abbreviations: RT-response time.

We conducted a series of experiments to determine the *Q*_*s*_ and *P*_*v*_ responses to a 10-fold increase and decrease in both uplift rate (scenario 1.1) and precipitation (scenario 2.1)^4,16^ (Fig. 2). Additional experimental runs were conducted to test the sensitivity of model results to absolute forcing magnitude (2- and 5-fold-changes in uplift and precipitation rates, scenarios 1.2–1.3 and 2.2–2.3); source area erodibility (*K*) (scenarios 1.4–1.5 and 2.4–2.5); provenance weighting coefficients (*c*) (scenarios 1.6–1.7 and 2.6–2.7); and model time step, size, and geometry (scenarios 1.8–1.15 and 2.8–2.15) (Table DR1). All model results are presented in Supplemental Fig. 1.

Each experiment contains three phases (Fig. 2). (1) An initially flat model surface (0 m elevation) is seeded with small, random variations to allow a stream network to develop. Uplift is added to the surface at a rate of 1 mm/yr, and the model is run until topographic steady-state is achieved (~2–2.5 Myr). (2) The model is then subjected to a stepped increase and subsequent stepped decrease in either uplift rate or precipitation (Fig. 2). The time duration of each step of the initial phase is sufficiently long to allow the model to reach steady-state (Fig. 2). We follow^4,16^ in defining the signal response time (*Q*_*s*_ or *P*_*v*_) as the time elapsed to return to within a threshold of the steady-state value (here defined as within 1%). (3) Following a similar procedure as previous workers^16^, the final phase consists of periodic, stepped forcing increase and decrease where the duration of each cycle is a percentage (100%, 75%, 50%, 25%, and 10%) of the *Q*_*s*_ response time determined during the second phase of the experiment (Fig. 2). The model is allowed to reach dynamic equilibrium, a condition where the *Q*_*s*_ and *P*_*v*_ signals are identical to the preceding cycle^16^, before the forcing period is changed (3 to 8 forcing cycles; Fig. 2). Because *Q*_*s*_ response time can differ for increasing versus decreasing forcing, periods of high and low forcing values are not necessarily of equal duration (Fig. 1b). This asymmetry is most pronounced for changes in precipitation, where the *Q*_*s*_ and *P*_*v*_ response times are much longer for a decrease in precipitation versus an increase (Fig. 2a).

## Results

### Signal Response to Stepped Forcing

#### Uplift Rate

Given a stepped change in uplift rate, *Q*_*s*_ increases to a new equilibrium value (*Q*_*s_eq*_) such that *Q*_*s_eq*_ = *U*_*new*_*A*, where *U*_*new*_ is the new rate of uplift and *A* is the catchment area. Maximum (*Q*_*s_max*_) and minimum (*Q*_*s_min*_) values are thus attained when the model is at steady-state during high and low uplift rates, respectively. In this case, *Q*_*s_min*_ ≈ 6 m^3^/yr and *Q*_*s_max*_ ≈ 60 m^3^/yr, with a response time of ~0.93 Myr for both increased and decreased uplift rate (Figs 1b and 2; Table DR2).

Unlike *Q*_*s*_, *P*_*v*_ does not tend towards a new equilibrium value, but instead exhibits a transient behavior where relative proximal erosion increases during an increase in uplift rate (peak change in *P*_*vA*_, *P*_*vA_max*_ ≈ 70% Source A) and relative distal erosion increases during a decrease in uplift rate (*P*_*vA_min*_ ≈ 38% Source A), before returning to the 50% equilibrium value that reflects the areal distribution of rock types A and B (Figs 1b and 2). The lag time between uplift rate change and *P*_*vA_max*_ is ~0.2 Myr, which is considerably shorter than the lag time of *Q*_*s_max*_ (~0.9 Myr). *P*_*v*_ also exhibits an asymmetry in response time: ~0.9 Myr for uplift rate increase and ~1.2 Myr for uplift rate decrease (Figs 1b and 2a).

#### Precipitation

As noted previously^4^, an increase in precipitation results in a rapid but temporary increase in *Q*_*s*_ followed by a return to *Q*_*s_eq*_. In our model, *Q*_*s_max*_ (19 m^3^/yr) is attained rapidly with a response time of ~0.3 Myr. During a decrease in precipitation, *Q*_*s_min*_ (~2 m^3^/yr) is also achieved rapidly but with a more prolonged response time (~0.9 Myr).

The *P*_*v*_ signal responds to changes in precipitation in an opposite way as to changes in uplift rate; distal erosion increases (*P*_*vA_max*_ ≈ 42% Source A) during precipitation increase and relative proximal erosion increases (*P*_*vA_max*_ ≈ 61% Source A) during precipitation decrease (Fig. 2). The lag time between precipitation increase and *P*_*vA_max*_ is ~0.2 Myr, whereas the lag time associated with a decrease in precipitation is longer (~0.6 Myr). The *P*_*v*_ response times for a precipitation increase and decrease (~0.4 Myr and ~0.9 Myr, respectively) are both less than the equivalent values for changing uplift rate (Fig. 2).

### Signal Response to Periodic Forcing

**Figure 3:**
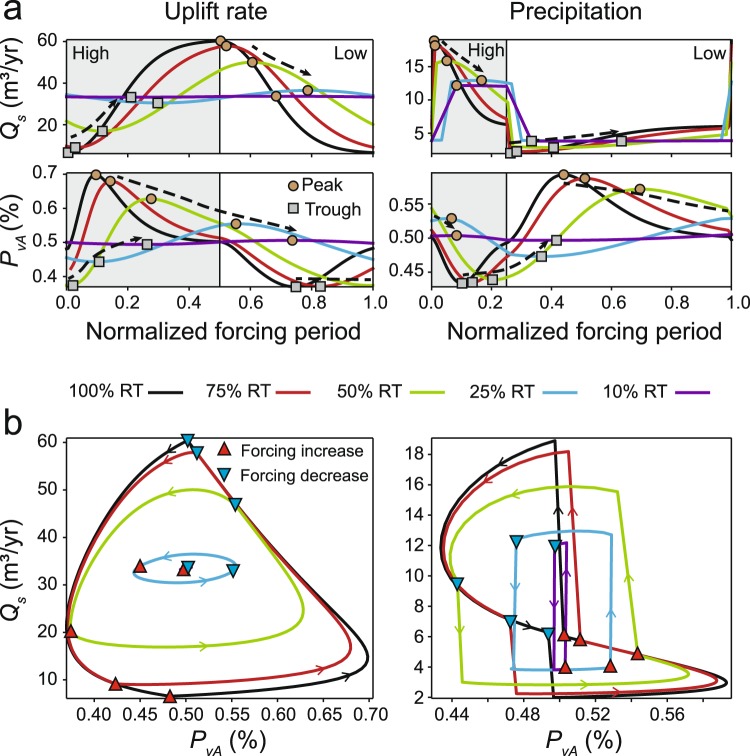
Sedimentary signal (*Q*_*s*_ and *P*_*vA*_) response in dynamic equilibrium (data position are shown in Fig. 2a). (**a**) Signal response normalized to forcing period. Dashed, black arrows indicate general direction of peak and trough translation as response time (RT) decreases. (**b**) *Q*_*s*_ versus *P*_*vA*_.

Our results corroborate previous research that suggests the *Q*_*s*_ signal is progressively modified as forcing periodicity becomes less than the *Q*_*s*_ response time^3,14,16^ and demonstrate that a similar pattern is characteristic of the *P*_*v*_ signal (Fig. 3).

#### Uplift Rate

As the periodicity of changing uplift rate becomes less than the *Q*_*s*_ response time, the relative amplitude of the *Q*_*s*_ signal is progressively damped such that the signal amplitude is <~10% of the maximum possible signal (*Q*_*s_max*_ − *Q*_*s_min*_) when the forcing period is 25% of the response time (Fig. 3a; Table DR3). At the same time, the relative timing of the peak *Q*_*s*_ migrates forward such that the peak is increasingly offset from when uplift rate increased (Fig. 3a). As noted by others^16^, the *Q*_*s*_ peak actually coincides with periods of low uplift rate for all scenarios where forcing periodicity is less than the *Q*_*s*_ response time.

Similar to the behavior of the *Q*_*s*_ signal, the amplitude of *P*_*v*_ is damped and the peak timing of *P*_*v*_ becomes increasingly offset as the forcing periodicity decreases (Fig. 3a). However, unlike *Q*_*s*_, the *P*_*vA*_ peak does coincide with the time of high uplift rate for scenarios with forcing periods >50% of the response time (Fig. 3a).

#### Precipitation

Model results show comparatively less damping of the *Q*_*s*_ signal as precipitation forcing periodicity decreases (Fig. 3). For example, when forcing periodicity is 25% of response time, variability in *Q*_*s*_ is still ~55% of its maximum variability (*Q*_*s_max*_ − *Q*_*s_min*_) achieved during the stepped forcing experiment. The behavior of the *P*_*v*_ signal in dynamic equilibrium under changing precipitation is similar to the general pattern observed under changing uplift rate; *P*_*v*_ amplitude is damped and peak *P*_*v*_ signal is increasingly offset from the timing of forcing onset (Fig. 3). The shape of the *Q*_*s*_ versus *P*_*v*_ response becomes increasingly rectilinear as forcing period decreases as a consequence of *Q*_*s*_ responding more rapidly than *P*_*v*_ to a change in precipitation rate (Fig. 3b).

### Sensitivity Analysis

The results from our sensitivity analysis (Scenarios 1.2–1.15 and 2.2–2.15) demonstrate similar *Q*_*s*_ and *P*_*v*_ signal responses to the base case under a range of model parameters (Table DR1 and Supplemental Fig. 1). The only significant exception is where the erodibility of the distal sediment source (Source B) is greater than the proximal sediment source (Source A) (Scenarios 1.5 and 2.5). In this case, the *P*_*v*_ signal includes both a peak and trough following a stepped change in forcing (Supplemental Fig. 1).

## Discussion

**Figure 4:**
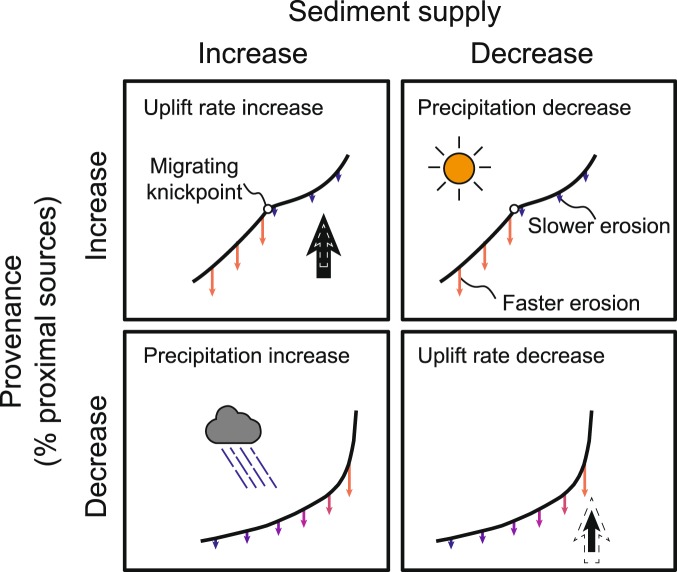
Illustration of how changes in *P*_*v*_ (proximal erosion) and *Q*_*s*_ are predicted to differentiate geomorphic response (knickpoint migration versus lowering of channel profile gradient) and environmental forcing (uplift rate versus precipitation).

Our results show that *Q*_*s*_ and *P*_*v*_ can have a variable response to the same change in forcing mechanism (Fig. 2). For instance, a plot of *Q*_*s*_ versus *P*_*vA*_ under dynamic equilibrium reveals strongly non-linear trends between these two sedimentary signals (Fig. 3b and Supplemental Fig. 1). The paired *Q*_*s*_ − *P*_*v*_ response is distinct between changes in uplift rate and precipitation, suggesting that these forcing mechanisms can be differentiated from each other (Figs 3, 4). Although an increase in uplift rate and precipitation will both produce an increase in *Q*_*s*_, the former is predicted to produce an increase in proximal erosion whereas the latter will produce an increase in distal erosion (Fig. 4). Similarly, although both increasing uplift rate and decreasing precipitation will increase proximal erosion, they will produce opposite trends in *Q*_*s*_ (Figs 2, 4). Because *Q*_*s*_ = *UA* at steady-state, a stepped change in uplift rate will produce a permanent change in *Q*_*s*_, whereas a stepped change in precipitation will produce only a transient change in *Q*_*s*_^15,17^. These findings are consistent with previous research based on analog modeling that has suggested that tectonic and climatic forcings can be differentiated by knowledge of both *Q*_*s*_ and mean catchment elevation^17^. Successful application of the paired *Q*_*s*_ − *P*_*v*_ response (Fig. 4) in natural systems will likely depend on the extent to which (1) *Q*_*s*_ can be estimated^18^, and (2) distinct rock types exist in the erosional catchment area. Identification of relative increases in proximal or distal erosion will be facilitated in circumstances where any contrasts in bedrock type are normal to the catchment, such that distal (high elevation) sediment sources yield distinct *P*_*v*_ versus proximal (low elevation) sources.

Further differentiation of tectonic versus climatic forcing mechanisms may be possible via analysis of the relative timing between peak *Q*_*s*_ and *P*_*v*_ responses. The *Q*_*s*_ response to precipitation change is predicted to be more rapid than the *P*_*v*_ response, with the opposite pattern for a change in uplift rate. Thus, our model results suggest that *P*_*v*_ may be a more sensitive signal of the timing of change in uplift rate, whereas *Q*_*s*_ is more sensitive to the timing of a change in precipitation. Signal response time also depends on forcing type and sign (increase or decrease). An increase in precipitation results in a signal (*Q*_*s*_ and *P*_*v*_) that peaks and then decreases very quickly with a typical response time of ~0.4 Myr. However, a decrease in precipitation results in a much longer signal response time (~0.9 Myr), and changes in uplift rate may produce signals that have typically longer response times (0.9–1.2 Myr).

The divergent responses of *Q*_*s*_ and *P*_*v*_ suggest differing geomorphic drivers behind these sedimentary signals. For instance, our modeling results predict that relative proximal erosion should increase in response to upstream knickpoint migration, a scenario that can occur in response to an increase in uplift rate or decrease in precipitation^28,29^. An increase in both proximal erosion and *Q*_*s*_ has been associated with an increase in normal fault uplift rate in the Central Apennines of Italy^10^. In this case, the region of increased proximal erosion was associated with a zone of pronounced convexity downstream of the knickpoint, as predicted by our model results^10^ (Fig. 4). Our model further predicts that distal erosion is favored in circumstances where streams incise to form lower gradient profiles, such as occur during a decrease in uplift rate or increase in precipitation^17^ (Fig. 4).

Although the modeling framework presented herein provides a first-order prediction of how the paired *Q*_*s*_ − *P*_*v*_ signal responds to tectonic and climatic forcings (Figs 3, 4), there are a number of potential caveats to applying model results to natural landscapes. (1) The stream-power erosional model (Equation 2) used herein does not allow for landsliding, a process that is known to exert an important control on sediment load and signal propagation in natural systems^30^. (2) Modeled precipitation changes does not account for variations in precipitation event intensity or frequency. For example, development of a drier, but stormier, climate may buffer the predicted *Q*_*s*_ and *P*_*v*_ response^31,32^. (3) Variations in bedrock erodibility may influence the *P*_*v*_ response, particularly when distal sources are more easily eroded than proximal sources (e.g., scenarios 1.5 and 2.5; Supplemental Fig. 1) and possibly in layered stratigraphy^33^. (4) *P*_*v*_ may also be sensitive to geomorphic processes not modeled herein, such as drainage divide migration or stream capture^34,35^.

Our results suggest that integration of multiple sedimentary parameters may provide greater resolving power than any single parameter alone (e.g. ref.^20^). Although *Q*_*s*_, grain size, and now *P*_*v*_ have been investigated in the context of sedimentary signal propagation, future efforts to characterize the relationship between geomorphic processes, their underlying physical mechanisms^36^, and resulting sediment characteristics may permit multi-dimensional analysis of signal transmission and propagation. For example, climate signals are hypothesized to be manifested as changes in sediment composition, such as the chemical index of alteration^37^, and there is opportunity for more research to elucidate how a sediment composition or weathering signal is transmitted from erosional landscapes undergoing changing forcing mechanisms. Thus, to the extent that sedimentary signals are produced by different geomorphic processes that operate at distinct, characteristic timescales, use of multiple sedimentary signals with distinct origins may greatly facilitate inversion of the stratigraphic record.

## Supplementary information

Supplementary materials

Animation - Scenario 1.1

Animation - Scenario 2.1
