# Author Correction: Conversion of tectonic and climatic forcings into records of sediment supply and provenance

**DOI:** 10.1038/s41598-020-75739-6

**Published:** 2020-11-02

**Authors:** Glenn R. Sharman, Zoltan Sylvester, Jacob A. Covault

**Affiliations:** 1 Department of Geosciences, University of Arkansas, Fayetteville, AR 72701 USA; 2 Bureau of Economic Geology, Jackson School of Geosciences, The University of Texas at Austin, Austin, TX 78758 USA

Correction to: *Scientific Reports* 10.1038/s41598-019-39754-6, published online 11 March 2019

This Article contains a repeated error, where the Q_s_ variable is not scaled by a factor of 10^4^. As a result, in the Results section under the subheading ‘Uplift Rate’,

“In this case, *Q*_*s_min*_ ≈ 6 m^3^/yr and *Q*_*s_max*_ ≈ 60 m^3^/yr, with a response time of ~ 0.93 Myr for both increased and decreased uplift rate (Figs 1b and 2; Table DR2).”

should read:

“In this case, *Q*_*s_min*_ ≈ 6 × 10^4^ m^3^/yr and *Q*_*s_max*_ ≈ 60 × 10^4^ m^3^/yr, with a response time of ~ 0.93 Myr for both increased and decreased uplift rate (Figs 1b and 2; Table DR2).”

In the Results section under the subheading ‘Precipitation’,

“In our model, *Q*_*s_max*_ (19 m^3^/yr) is attained rapidly with a response time of ~ 0.3 Myr. During a decrease in precipitation, *Q*_*s_min*_ (~ 2 m^3^/yr) is also achieved rapidly but with a more prolonged response time (~ 0.9 Myr).”

should read:

“In our model, *Q*_*s_max*_ (19 × 10^4^ m^3^/yr) is attained rapidly with a response time of ~ 0.3 Myr. During a decrease in precipitation, *Q*_*s_min*_ (~ 2 × 10^4^ m^3^/yr) is also achieved rapidly but with a more prolonged response time (~ 0.9 Myr).”

In Figure 1 in panel (b), the y-axis label,

“Q_s_ (m^3^yr^-1^)”

should read:

“Q_s_ (× 10^4^ m^3^yr^−1^)”.

In Figure 3 in panel (a-b), the y-axis label,

“Q_s_ (m^3^/yr)”

should read:

“Q_s_ (× 10^4^ m^3^yr^−1^)”.

In Supplementary Table DR2, the units for ‘Magnitude of Change’ for both ‘Q_s_min_’ and ‘Q_s_max_’,

“(m^3^yr^−1^)”

should read:

“(× 10^4^ m^3^yr^-1^)”.

In Supplemental Figure 1, for all Scenarios, the y-axis label,

“Q_s_ (m^3^/yr)”

should read:

“Q_s_ (× 10^4^ m^3^/yr)”.

In Supplementary Scenarios 1.1-1.15, Scenarios 2.1-2.15, and Animations, in the graph ‘Change in Qs and provenance (% Source A) over time’, the unit is omitted for the y-axis, where

“Q_s_”

should read:

“Q_s_ (× 10^4^ m^3^/yr)”.

