# Supplementary material for: Conversion of tectonic and climatic forcings into records of sediment supply and provenance
Source: Sci Rep. 2019 Mar 11;9:4115. doi: 10.1038/s41598-019-39754-6 (PMC6411995; doi:10.1038/s41598-019-39754-6)

Conversion of tectonic and climatic forcings into records of sediment supply and provenance

Glenn R. Sharman, Zoltan Sylvester, and Jacob A. Covault

Supplementary Materials

Summary of Contents

Table DR1: Model Parameters

Table DR2: Analysis of Stepped Change in Forcing

Table DR3: Analysis of Periodic Change in Forcing

Supplemental Figures 1 & 2: Experimental results for all model runs

Animations (Scenario 1.1 & 2.1)

Table DR1. Model Parameters

Scenario	Uplift rate (m/yr)	Rainfall intensity	Timestep (yr)	K ($m^{1-2m}yr^{-1}$)	c	A:B*	Rows	Columns	Gridcell (m)
1.1 (base case)	0.001 to 0.01	1	10,000	0.00001	1	1:1	80	80	100
1.2	0.001 to 0.005	1	10,000	0.00001	1	1:1	80	80	100
1.3	0.001 to 0.002	1	10,000	0.00001	1	1:1	80	80	100
1.4	0.001 to 0.01	1	10,000	0.00002(A), 0.00001(B)	1	1:1	80	80	100
1.5	0.001 to 0.01	1	10,000	0.00001(A), 0.00002(B)	1	1:1	80	80	100
1.6	0.001 to 0.01	1	10,000	0.00001	2(A), 1(B)	1:1	80	80	100
1.7	0.001 to 0.01	1	10,000	0.00001	1(A), 2(B)	1:1	80	80	100
1.8	0.001 to 0.01	1	1,000	0.00001	1	1:1	80	80	100
1.9	0.001 to 0.01	1	10,000	0.00001	1	1:1	160	160	100
1.10	0.001 to 0.01	1	10,000	0.00001	1	1:1	160	80	100
1.11	0.001 to 0.01	1	10,000	0.00001	1	2:1	160	80	100
1.12	0.001 to 0.01	1	10,000	0.00001	1	1:2	160	80	100
1.13	0.001 to 0.01	1	10,000	0.00001	1	1:1	80	160	100
1.14	0.001 to 0.01	1	10,000	0.00001	1	2:1	80	160	100
1.15	0.001 to 0.01	1	10,000	0.00001	1	1:2	80	160	100
2.1 (base case)	0.001	1 to 10	10,000	0.00001	1	1:1	80	80	100
2.2	0.001	1 to 5	10,000	0.00001	1	1:1	80	80	100
2.3	0.001	1 to 2	10,000	0.00001	1	1:1	80	80	100
2.4	0.001	1 to 10	10,000	0.00002(A), 0.00001(B)	1	1:1	80	80	100
2.5	0.001	1 to 10	10,000	0.00001(A), 0.00002(B)	1	1:1	80	80	100
2.6	0.001	1 to 10	10,000	0.00001	1(A), 0.5(B)	1:1	80	80	100
2.7	0.001	1 to 10	10,000	0.00001	0.5(A), 1(B)	1:1	80	80	100
2.8	0.001	1 to 10	1,000	0.00001	1	1:1	80	80	100
2.9	0.001	1 to 10	10,000	0.00001	1	1:1	160	160	100
2.10	0.001	1 to 10	10,000	0.00001	1	1:1	160	80	100
2.11	0.001	1 to 10	10,000	0.00001	1	2:1	160	80	100
2.12	0.001	1 to 10	10,000	0.00001	1	1:2	160	80	100
2.13	0.001	1 to 10	10,000	0.00001	1	1:1	80	160	100
2.14	0.001	1 to 10	10,000	0.00001	1	2:1	80	160	100
2.15	0.001	1 to 10	10,000	0.00001	1	1:2	80	160	100

Notes: (A) indicates property is assigned to Source A and (B) indicates property is assigned to Source B

*A:B = the ratio of aerial extents of Source A to Source B

In all scenarios, m=0.5 and n=1 (see Equation 2 of manuscript text)

Table DR2. Analysis of Stepped Change in Forcing

Scenario*	Increased forcing		Decreased forcing		Magnitude of Change				Max	Max Erosion
	Q_s RT[†] (yr)	P_v RT (yr)	Q_s RT (yr)	P_v RT (yr)	Q_{s_min} (m^3yr^{-1})	Q_{s_max} (m^3yr^{-1})	P_{v_min} (%)	P_{v_max} (%)	Elevation (m)	Rate (m/yr)
1.1	930,000	840,000	930,000	1,140,000	6.1	60.8	38.2	70.4	8392	0.0178
1.2	930,000	880,000	930,000	1,100,000	6.1	30.4	39.5	64.1	4148	0.00849
1.3	950,000	950,000	950,000	1,070,000	6.1	12.2	44.9	55.8	1659	0.00479
1.4	840,000	800,000	830,000	1,080,000	6.1	60.8	18.8	78.9	7350	0.0178
1.5	780,000	850,000	770,000	1,180,000	6.1	60.9	44.6	66.4	7136	0.0173
1.6	940,000	900,000	930,000	1,150,000	6.1	60.9	52.7	83.5	8026	0.0169
1.7	930,000	900,000	930,000	1,150,000	6.1	60.8	20.9	56.8	8558	0.0173
1.8	888,000	834,000	888,000	1,075,000	6.1	60.8	35.7	73.1	7725	0.0188
1.9	1,020,000	1,000,000	1,020,000	1,000,000	25	250	32.7	73.1	10201	0.0321
1.10	1,000,000	980,000	1,000,000	1,230,000	12.3	123	31.9	73.1	9988	0.0207
1.11	1,000,000	1,020,000	1,010,000	1,250,000	12.3	123	47.6	86.7	10567	0.0303
1.12	1,000,000	960,000	1,000,000	1,190,000	12.3	123	18.70	56.60	9268	0.0286
1.13	950,000	910,000	960,000	1,170,000	12.3	123	38.8	69.3	8740	0.0214
1.14	950,000	1,020,000	950,000	1,090,000	12.3	123	56.7	83.7	8447	0.0193
1.15	970,000	890,000	960,000	1,180,000	12.3	123	24.3	50.0	8949	0.0167
2.1	300,000	360,000	920,000	940,000	1.9	19.1	42.0	60.7	972	0.00518
2.2	410,000	510,000	910,000	990,000	2.7	13.6	43.8	57.2	962	0.00433
2.3	610,000	700,000	910,000	1,010,000	4.3	9	47.3	52.9	960	0.00445
2.4	270,000	330,000	810,000	830,000	1.9	18.7	34.7	66.0	871	0.00447
2.5	240,000	360,000	750,000	930,000	1.9	19.1	46.9	54.8	767	0.00546
2.6	300,000	340,000	920,000	1,170,000	1.9	19.1	59.6	75.0	1002	0.00573
2.7	300,000	360,000	920,000	940,000	1.9	19.1	26.8	43.1	991	0.00483
2.8	279,000	309,000	895,000	1,043,000	1.9	19.3	40.3	61.6	1110	0.00856
2.9	330,000	380,000	1,000,000	1,130,000	7.9	78.7	41.3	60.0	1560	0.00939
2.10	320,000	380,000	990,000	1,230,000	3.9	38.8	41.3	60.2	1552	0.00838
2.11	320,000	380,000	980,000	1,130,000	3.9	38.8	57.2	77.5	1480	0.00857
2.12	330,000	390,000	1,010,000	1,030,000	3.9	38.8	25.7	42.7	1473	0.00902
2.13	310,000	360,000	930,000	910,000	3.9	38.7	44.9	57.6	1071	0.00593
2.14	310,000	370,000	940,000	980,000	3.9	38.8	60.4	75.8	1112	0.00586
2.15	310,000	360,000	950,000	990,000	3.9	38.7	28.0	40.2	1089	0.00567

*See Table DR1 for an explanation of parameters used in each scenario

[†]RT = Response time to return to 99% of equilibrium value (see text for explanation)

Abbreviations: incr: increase; decr: decrease.

Table DR3. Analysis of Periodic Changes in Forcing

Scenario*	Forcing period (RT[†]%)	Q_{s_min} lag (%)[§]	Q_{s_max} lag (%)[§]	P_{v_min} lag (%)[§]	P_{v_max} peak lag (%)[§]	ΔQ_s (%)[#]	ΔP_v (%)[#]
1.1	100%	0.5%	50.5%	74.7%	9.1%	98.3%	95.0%
1.1	75%	2.9%	52.3%	83.2%	14.3%	89.4%	89.3%
1.1	50%	11.8%	61.3%	1.1%	26.9%	60.6%	73.3%
1.1	25%	32.3%	79.6%	8.6%	55.9%	10.9%	31.7%
1.1	10%	16.1%	64.5%	21.5%	69.9%	0.9%	4.1%
1.2	100%	0.5%	50.5%	72.6%	11.3%	98.0%	98.2%
1.2	75%	2.9%	52.3%	80.3%	16.5%	88.6%	94.9%
1.2	50%	11.8%	61.3%	95.7%	30.1%	59.2%	82.2%
1.2	25%	32.3%	81.7%	10.8%	58.1%	10.0%	36.4%
1.2	10%	16.1%	64.5%	21.5%	69.9%	0.8%	4.2%
1.3	100%	0.5%	50.5%	68.4%	13.7%	98.2%	98.4%
1.3	75%	2.1%	51.9%	74.4%	18.9%	89.5%	93.8%
1.3	50%	11.6%	61.1%	88.4%	31.6%	59.5%	84.0%
1.3	25%	31.6%	80.0%	12.6%	58.9%	9.1%	41.4%
1.3	10%	26.3%	73.7%	31.6%	78.9%	1.0%	3.7%
1.4	100%	0.6%	50.9%	70.7%	9.0%	98.2%	98.7%
1.4	75%	0.8%	51.1%	78.2%	12.8%	91.1%	94.9%
1.4	50%	3.6%	52.7%	92.2%	25.1%	66.6%	83.9%
1.4	25%	9.6%	59.9%	7.2%	55.1%	28.5%	47.6%
1.4	10%	24.0%	71.9%	24.0%	71.9%	4.3%	6.5%
1.5	100%	0.6%	51.0%	0.6%	7.1%	98.4%	74.2%
1.5	75%	3.4%	53.3%	31.0%	9.5%	91.9%	74.7%
1.5	50%	10.3%	60.6%	46.5%	12.9%	71.6%	88.4%
1.5	25%	33.5%	82.6%	90.3%	38.7%	22.0%	53.6%
1.5	10%	12.9%	58.1%	32.3%	83.9%	0.9%	3.7%
1.6	100%	0.5%	50.8%	75.9%	10.2%	98.1%	98.7%
1.6	75%	3.6%	52.8%	84.8%	15.7%	88.6%	94.5%
1.6	50%	10.7%	61.0%	1.1%	28.9%	58.9%	81.4%
1.6	25%	29.9%	79.1%	10.7%	59.9%	9.7%	32.4%
1.6	10%	21.4%	69.5%	26.7%	74.9%	0.9%	2.9%
1.7	100%	0.5%	50.5%	75.3%	10.2%	98.0%	97.8%
1.7	75%	3.6%	53.0%	83.9%	15.8%	88.0%	90.6%
1.7	50%	11.8%	61.3%	2.2%	29.0%	57.7%	71.8%
1.7	25%	30.1%	79.6%	10.8%	58.1%	9.6%	30.6%
1.7	10%	21.5%	69.9%	32.3%	80.6%	0.9%	2.5%

Table DR3. Analysis of Periodic Changes in Forcing

Scenario*	Forcing period (RT[†]%)	Q_{s_min} lag (%)[§]	Q_{s_max} lag (%)[§]	P_{v_min} lag (%)[§]	P_{v_max} peak lag (%)[§]	ΔQ_s (%)[#]	ΔP_v (%)[#]
1.8	100%	0.1%	50.1%	75.0%	10.1%	98.1%	97.3%
1.8	75%	2.3%	52.3%	83.3%	14.8%	89.1%	92.8%
1.8	50%	11.0%	61.0%	99.9%	27.9%	59.9%	78.3%
1.8	25%	31.5%	81.5%	12.4%	60.1%	9.8%	32.6%
1.8	10%	22.5%	70.9%	28.2%	77.7%	0.7%	3.0%
1.9	100%	0.5%	50.5%	77.5%	11.3%	98.0%	97.5%
1.9	75%	4.6%	54.2%	86.9%	17.0%	88.0%	92.7%
1.9	50%	12.7%	62.7%	1.0%	31.4%	58.0%	77.8%
1.9	25%	35.3%	84.3%	17.6%	66.7%	8.3%	26.8%
1.9	10%	24.5%	73.5%	29.4%	78.4%	0.6%	1.8%
1.10	100%	0.5%	50.5%	77.5%	11.0%	97.7%	98.6%
1.10	75%	3.3%	53.3%	86.7%	16.7%	87.3%	93.5%
1.10	50%	12.0%	62.0%	1.0%	32.0%	56.3%	78.1%
1.10	25%	30.0%	80.0%	16.0%	64.0%	9.6%	27.8%
1.10	10%	25.0%	75.0%	35.0%	85.0%	0.7%	2.2%
1.11	100%	0.5%	50.2%	78.1%	13.4%	97.8%	98.4%
1.11	75%	3.3%	53.1%	87.6%	19.2%	87.6%	96.1%
1.11	50%	12.9%	62.7%	2.0%	34.8%	56.9%	83.2%
1.11	25%	31.8%	81.6%	23.9%	71.6%	9.6%	27.8%
1.11	10%	19.9%	69.7%	5.0%	54.7%	0.6%	0.7%
1.12	100%	0.5%	50.5%	75.0%	10.0%	98.3%	96.2%
1.12	75%	2.7%	52.7%	83.3%	14.7%	89.5%	90.3%
1.12	50%	12.0%	62.0%	0.0%	27.0%	60.4%	74.3%
1.12	25%	34.0%	84.0%	10.0%	58.0%	9.5%	30.1%
1.12	10%	20.0%	70.0%	15.0%	65.0%	0.8%	3.1%
1.13	100%	0.5%	50.3%	74.9%	9.9%	98.2%	98.9%
1.13	75%	2.8%	52.4%	82.4%	14.0%	89.0%	93.7%
1.13	50%	11.5%	60.7%	0.0%	27.2%	59.1%	77.0%
1.13	25%	31.4%	81.7%	10.5%	56.5%	9.3%	34.1%
1.13	10%	26.2%	73.3%	31.4%	78.5%	0.8%	3.0%
1.14	100%	0.5%	50.5%	76.3%	11.6%	98.1%	97.6%
1.14	75%	2.8%	52.6%	84.9%	16.1%	88.8%	93.3%
1.14	50%	13.7%	63.2%	2.1%	31.6%	58.7%	78.9%
1.14	25%	35.8%	84.2%	18.9%	65.3%	8.3%	29.5%
1.14	10%	26.3%	73.7%	36.8%	84.2%	0.7%	1.0%

Table DR3. Analysis of Periodic Changes in Forcing

Scenario*	Forcing period (RT[†]%)	Q_{s_min} lag (%)[§]	Q_{s_max} lag (%)[§]	P_{v_min} lag (%)[§]	P_{v_max} peak lag (%)[§]	ΔQ_s (%)[#]	ΔP_v (%)[#]
1.15	100%	0.5%	50.8%	73.6%	8.3%	98.3%	96.8%
1.15	75%	2.8%	52.5%	80.8%	11.7%	89.4%	89.4%
1.15	50%	12.4%	62.2%	96.4%	23.8%	60.3%	69.0%
1.15	25%	35.2%	85.0%	4.1%	51.8%	9.5%	32.6%
1.15	10%	20.7%	72.5%	15.5%	62.2%	0.7%	6.5%
2.1	100%	25.4%	0.8%	9.8%	43.4%	99.0%	97.8%
2.1	75%	27.3%	1.1%	13.1%	50.3%	94.1%	94.0%
2.1	50%	39.3%	4.9%	21.3%	68.9%	78.2%	82.2%
2.1	25%	59.0%	9.8%	36.1%	3.3%	55.4%	35.5%
2.1	10%	41.0%	8.2%	49.2%	8.2%	50.5%	4.6%
2.2	100%	31.8%	0.8%	12.1%	50.0%	98.8%	98.5%
2.2	75%	34.3%	2.0%	17.2%	57.6%	93.5%	95.4%
2.2	50%	47.0%	7.6%	27.3%	75.8%	77.2%	83.7%
2.2	25%	66.7%	18.2%	45.5%	6.1%	54.3%	37.2%
2.2	10%	45.5%	7.6%	53.0%	7.6%	50.9%	4.5%
2.3	100%	40.8%	0.7%	15.1%	58.6%	98.5%	97.7%
2.3	75%	43.9%	2.6%	21.1%	65.8%	92.7%	95.2%
2.3	50%	53.9%	9.2%	32.9%	81.6%	76.4%	85.3%
2.3	25%	73.7%	23.7%	52.6%	10.5%	53.8%	37.7%
2.3	10%	59.2%	13.2%	65.8%	13.2%	50.9%	3.9%
2.4	100%	25.9%	0.9%	9.3%	41.7%	98.9%	98.9%
2.4	75%	27.2%	2.5%	13.6%	49.4%	95.0%	95.3%
2.4	50%	29.6%	3.7%	20.4%	66.7%	83.0%	83.2%
2.4	25%	37.0%	7.4%	33.3%	3.7%	65.4%	44.8%
2.4	10%	46.3%	9.3%	37.0%	0.0%	56.4%	6.9%
2.5	100%	25.3%	1.0%	2.0%	0.0%	98.8%	88.3%
2.5	75%	28.3%	1.3%	2.7%	17.5%	95.3%	85.8%
2.5	50%	38.4%	4.0%	2.0%	40.4%	83.2%	90.4%
2.5	25%	68.7%	12.1%	16.2%	76.8%	58.5%	50.9%
2.5	10%	30.3%	10.1%	30.3%	0.0%	52.1%	5.5%
2.6	100%	25.4%	0.8%	9.8%	43.4%	99.0%	95.7%
2.6	75%	27.3%	1.1%	13.1%	50.3%	94.2%	92.2%
2.6	50%	39.3%	4.9%	21.3%	70.5%	78.4%	81.5%
2.6	25%	62.3%	9.8%	36.1%	3.3%	55.3%	35.0%
2.6	10%	41.0%	8.2%	49.2%	8.2%	50.4%	4.1%

Table DR3. Analysis of Periodic Changes in Forcing

Scenario*	Forcing period (RT[†]%)	Q_{s_min} lag (%)[§]	Q_{s_max} lag (%)[§]	P_{v_min} lag (%)[§]	P_{v_max} peak lag (%)[§]	ΔQ_s (%)[#]	ΔP_v (%)[#]
2.7	100%	25.4%	0.8%	9.8%	43.4%	99.1%	98.3%
2.7	75%	27.3%	1.1%	13.1%	50.3%	94.7%	94.7%
2.7	50%	39.3%	4.9%	21.3%	68.9%	79.3%	82.9%
2.7	25%	62.3%	9.8%	36.1%	3.3%	55.6%	35.5%
2.7	10%	41.0%	8.2%	49.2%	8.2%	50.4%	4.5%
2.8	100%	23.9%	0.1%	9.9%	43.8%	98.8%	94.3%
2.8	75%	27.4%	1.0%	13.3%	51.4%	94.2%	92.1%
2.8	50%	41.1%	5.5%	20.4%	70.4%	79.2%	82.4%
2.8	25%	74.6%	15.7%	42.6%	5.5%	54.3%	36.7%
2.8	10%	41.7%	6.0%	66.4%	13.6%	50.9%	2.8%
2.9	100%	25.6%	0.8%	10.5%	45.9%	98.9%	96.3%
2.9	75%	29.1%	1.0%	14.0%	53.1%	94.1%	93.1%
2.9	50%	43.6%	6.0%	22.6%	73.7%	78.4%	82.2%
2.9	25%	72.2%	15.0%	45.1%	6.0%	54.2%	32.9%
2.9	10%	45.1%	7.5%	52.6%	7.5%	51.2%	2.6%
2.10	100%	25.2%	0.8%	10.7%	45.0%	98.8%	97.0%
2.10	75%	28.5%	1.0%	14.2%	52.9%	94.3%	94.3%
2.10	50%	41.2%	4.6%	21.4%	73.3%	78.6%	83.3%
2.10	25%	67.2%	15.3%	42.7%	3.1%	54.1%	32.4%
2.10	10%	45.8%	15.3%	53.4%	15.3%	49.9%	3.0%
2.11	100%	25.4%	0.8%	11.5%	48.5%	98.8%	93.8%
2.11	75%	29.7%	1.0%	15.4%	57.4%	93.5%	90.8%
2.11	50%	43.1%	6.2%	24.6%	80.0%	77.2%	80.6%
2.11	25%	67.7%	15.4%	52.3%	9.2%	53.5%	25.9%
2.11	10%	46.2%	15.4%	53.8%	15.4%	50.0%	1.3%
2.12	100%	25.4%	0.7%	9.7%	43.3%	99.1%	96.8%
2.12	75%	28.9%	2.0%	13.9%	50.7%	94.3%	93.1%
2.12	50%	43.3%	6.0%	22.4%	70.1%	78.2%	81.1%
2.12	25%	68.7%	14.9%	32.8%	3.0%	54.2%	35.9%
2.12	10%	37.3%	7.5%	37.3%	0.0%	51.5%	5.9%
2.13	100%	25.8%	0.8%	9.7%	43.5%	98.8%	98.7%
2.13	75%	30.1%	2.2%	14.0%	51.6%	93.5%	95.3%
2.13	50%	43.5%	6.5%	22.6%	71.0%	77.0%	82.2%
2.13	25%	67.7%	16.1%	38.7%	6.5%	54.5%	34.7%
2.13	10%	40.3%	8.1%	48.4%	8.1%	50.2%	4.5%

Table DR3. Analysis of Periodic Changes in Forcing

Scenario*	Forcing period (RT[†]%)	Q_{s_min} lag (%)[§]	Q_{s_max} lag (%)[§]	P_{v_min} lag (%)[§]	P_{v_max} peak lag (%)[§]	ΔQ_s (%)[#]	ΔP_v (%)[#]
2.14	100%	25.6%	0.8%	10.4%	45.6%	98.9%	99.1%
2.14	75%	28.8%	1.1%	13.9%	53.3%	94.0%	96.2%
2.14	50%	41.6%	4.8%	22.4%	73.6%	78.1%	84.4%
2.14	25%	70.4%	16.0%	48.0%	9.6%	54.6%	30.9%
2.14	10%	40.0%	8.0%	48.0%	8.0%	50.1%	2.0%
2.15	100%	25.4%	0.8%	9.5%	40.5%	99.0%	98.2%
2.15	75%	28.6%	1.1%	12.7%	46.6%	94.3%	94.0%
2.15	50%	42.9%	6.3%	20.6%	66.7%	77.9%	79.2%
2.15	25%	66.7%	15.9%	28.6%	3.2%	54.7%	37.5%
2.15	10%	39.7%	7.9%	31.7%	0.0%	50.2%	11.0%

*See Table DR1 for an explanation of parameters used in each scenario

[†]Forcing period is a percentage of the Q_s response time to stepped increase and decrease in forcing (see text for explanation)

[§]Lag time is normalized to forcing period (see Table DR2)

[#]Change in Q_s and P_v is normalized to maximum difference from stepped experiment (see Table DR2)

Supplemental Figure 1

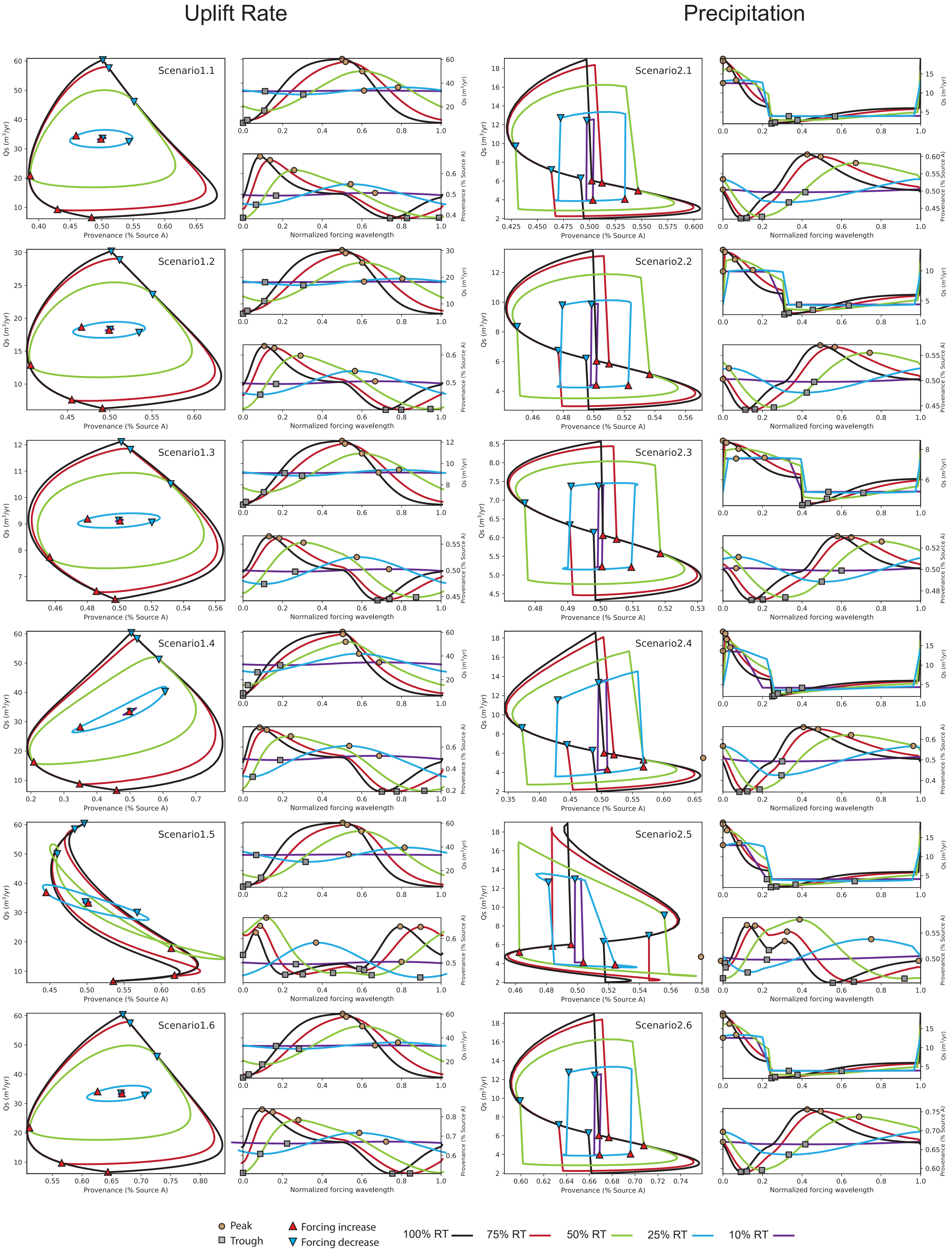

Supplemental Figure 1 (continued)

Uplift Rate

Precipitation

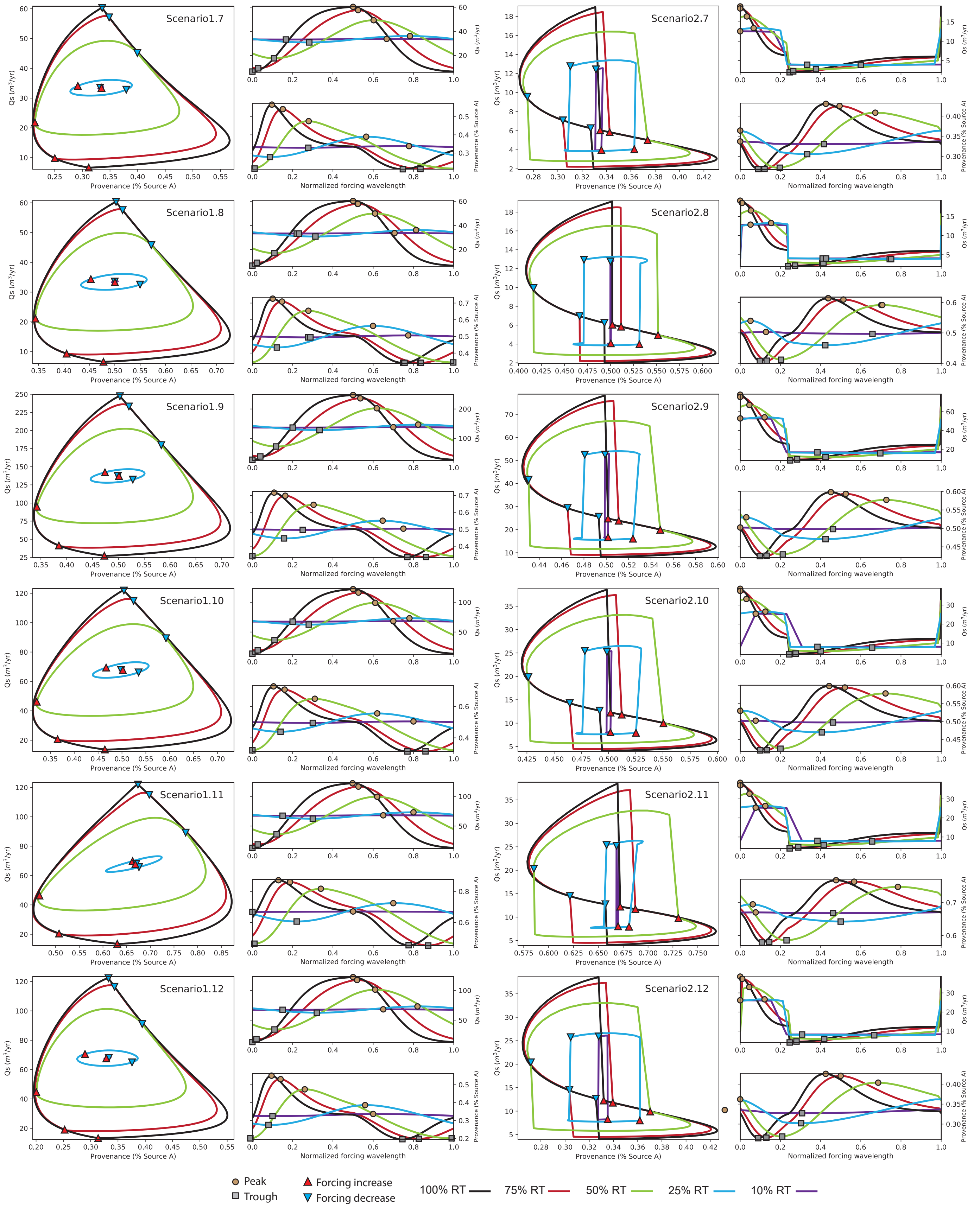

Supplemental Figure 1 (continued)

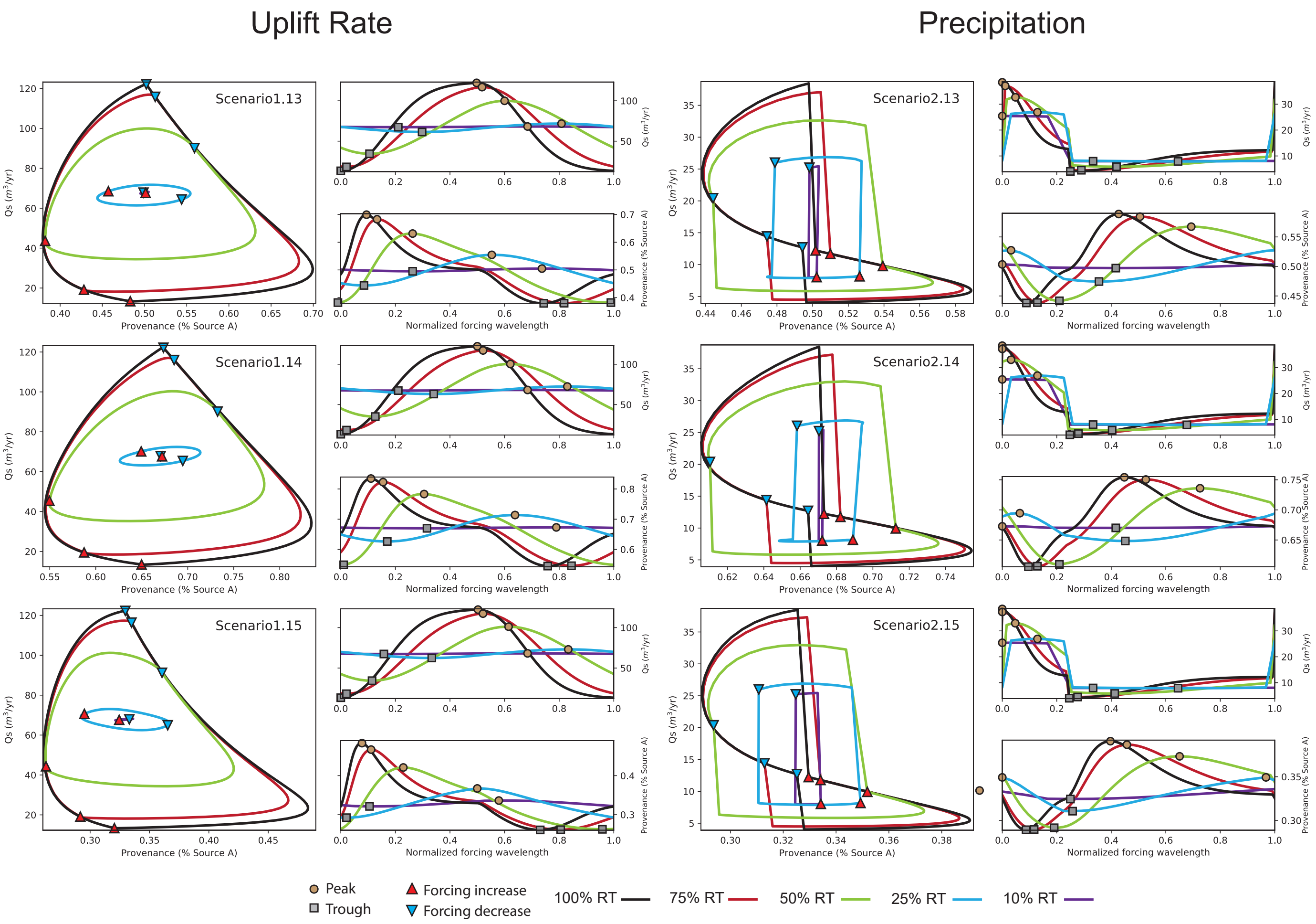

Supplemental Figure 1. Experimental results for all model runs (see Tables DR1 and DR3 for an explanation of model parameters and for dynamic equilibrium results, respectively). Final snapshots of all experimental runs are shown in Supplementary Figure 2. See Figure 3 and the main manuscript text for further explanation.

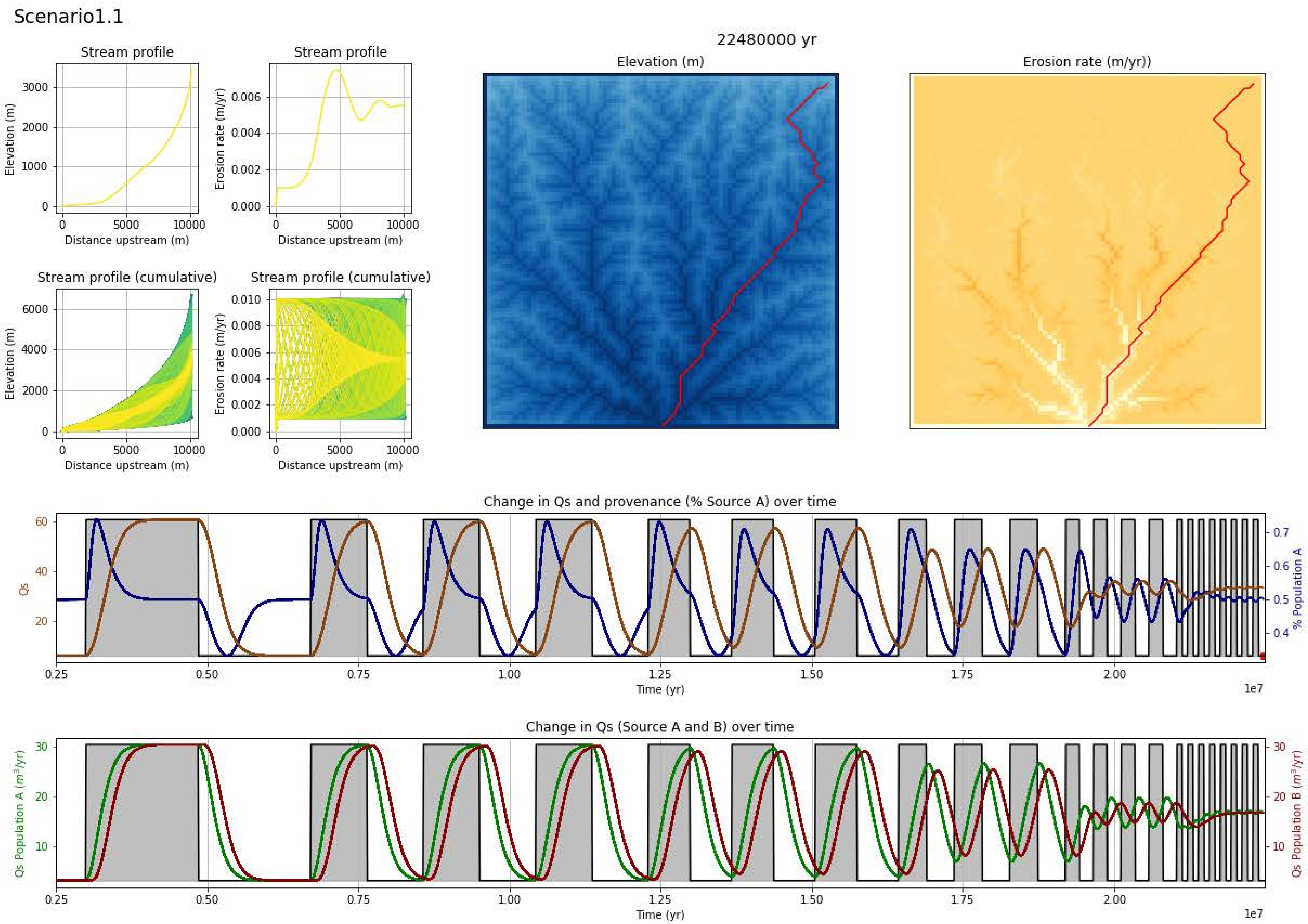

Scenario1.1
22480000 yr
Stream profile
Elevation (m)
Distance upstream (m)
Stream profile
Erosion rate (m/yr)
Distance upstream (m)
Stream profile (cumulative)
Elevation (m)
Distance upstream (m)
Stream profile (cumulative)
Erosion rate (m/yr)
Distance upstream (m)
Elevation (m)
Erosion rate (m/yr))
Change in Qs and provenance (% Source A) over time
Qs
% Population A
Time (yr)
1e7
Change in Qs (Source A and B) over time
Qs Population A (m³/yr)
Qs Population B (m³/yr)
Time (yr)
1e7

Scenario1.2

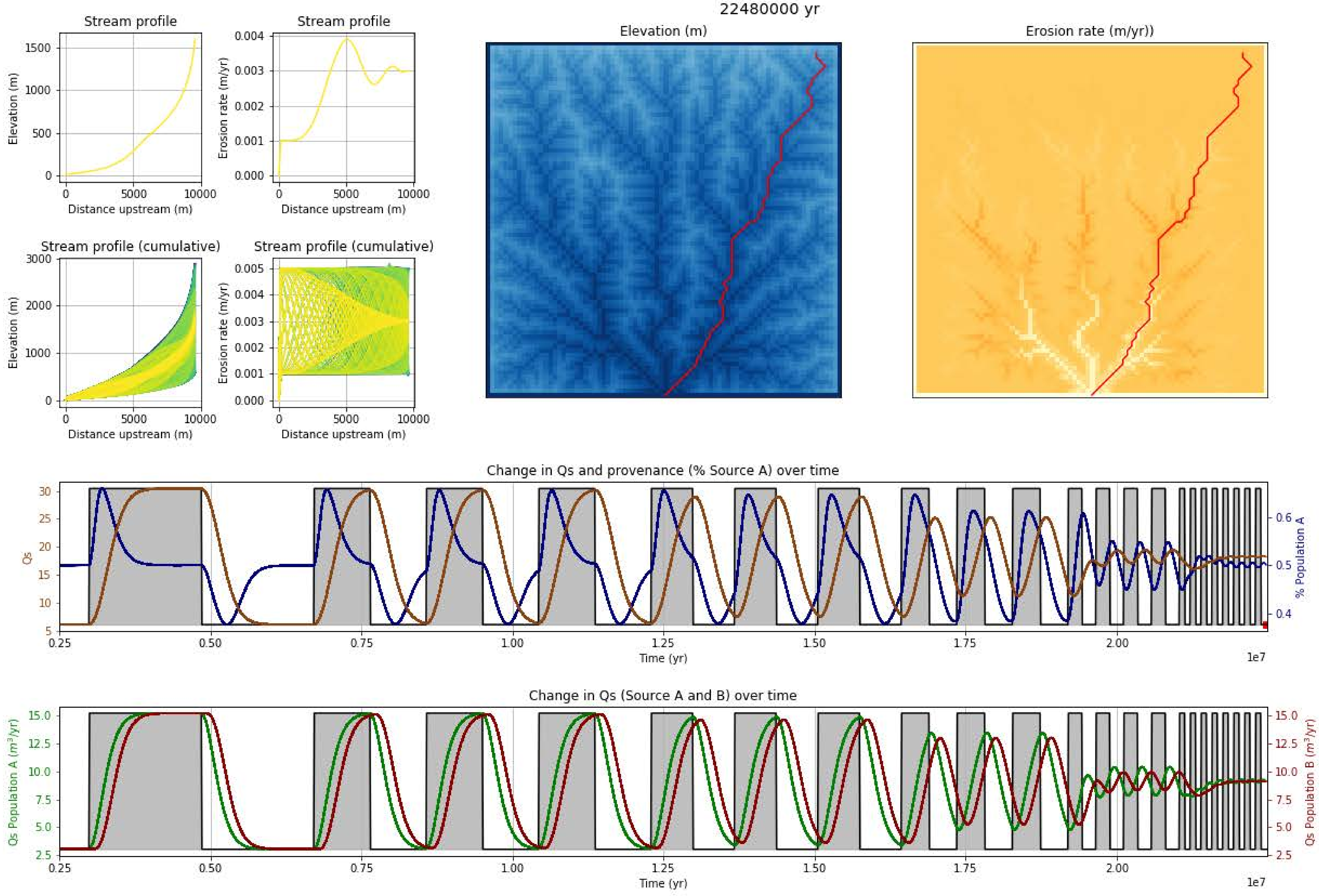

22480000 yr
Stream profile
Elevation (m)
Distance upstream (m)
Stream profile
Erosion rate (m/yr)
Distance upstream (m)
Stream profile (cumulative)
Elevation (m)
Distance upstream (m)
Stream profile (cumulative)
Erosion rate (m/yr)
Distance upstream (m)
Elevation (m)
Erosion rate (m/yr))
Change in Qs and provenance (% Source A) over time
Qs
% Population A
Time (yr)
1e7
Change in Qs (Source A and B) over time
Qs Population A (m^3/yr)
Qs Population B (m^3/yr)
Time (yr)
1e7

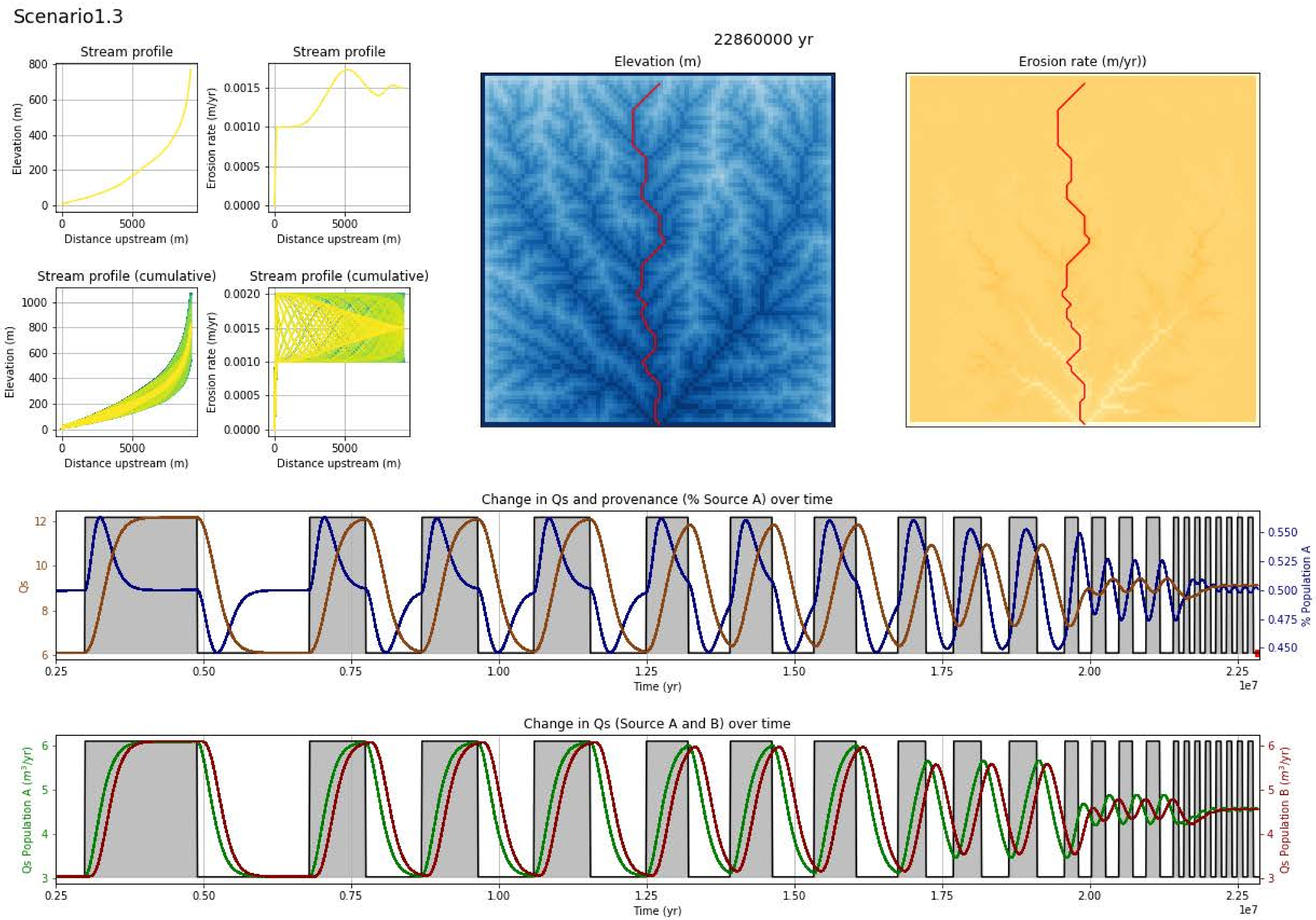

Scenario1.3
22860000 yr
Stream profile
Elevation (m)
Distance upstream (m)
Stream profile
Erosion rate (m/yr)
Distance upstream (m)
Stream profile (cumulative)
Elevation (m)
Distance upstream (m)
Stream profile (cumulative)
Erosion rate (m/yr)
Distance upstream (m)
Elevation (m)
Erosion rate (m/yr))
Change in Qs and provenance (% Source A) over time
Qs
% Population A
Time (yr)
1e7
Change in Qs (Source A and B) over time
Qs Population A (m³/yr)
Qs Population B (m³/yr)
Time (yr)
1e7

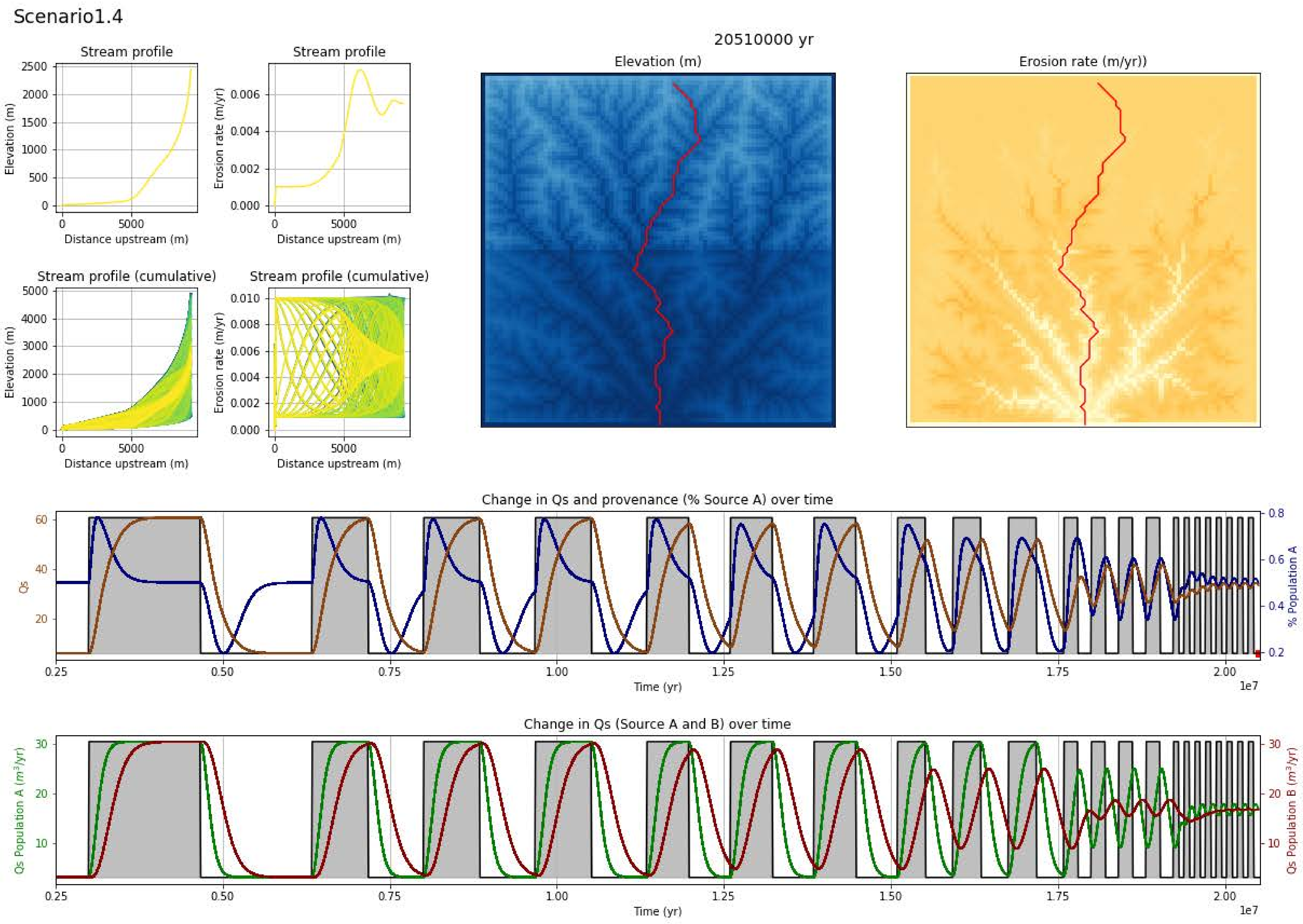
Scenario1.4
20510000 yr
Stream profile
Elevation (m)
Distance upstream (m)
Stream profile
Erosion rate (m/yr)
Distance upstream (m)
Stream profile (cumulative)
Elevation (m)
Distance upstream (m)
Stream profile (cumulative)
Erosion rate (m/yr)
Distance upstream (m)
Elevation (m)
Erosion rate (m/yr))
Change in Qs and provenance (% Source A) over time
Qs
% Population A
Time (yr)
1e7
Change in Qs (Source A and B) over time
Qs Population A (m^3/yr)
Qs Population B (m^3/yr)
Time (yr)
1e7

Scenario1.5

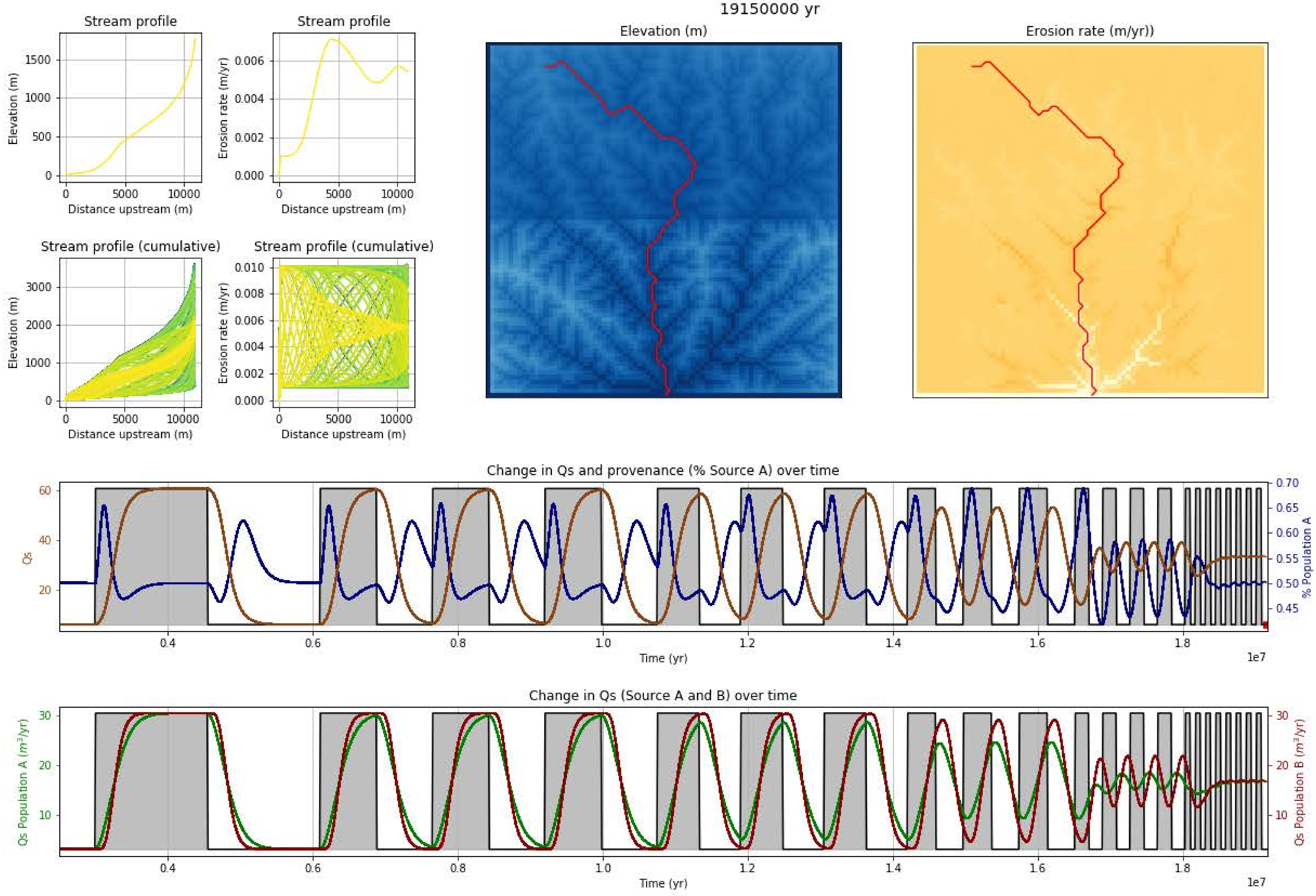
19150000 yr
Stream profile
Elevation (m)
Distance upstream (m)
Stream profile
Erosion rate (m/yr)
Distance upstream (m)
Stream profile (cumulative)
Elevation (m)
Distance upstream (m)
Stream profile (cumulative)
Erosion rate (m/yr)
Distance upstream (m)
Elevation (m)
Erosion rate (m/yr))
Change in Qs and provenance (% Source A) over time
Qs
% Population A
Time (yr)
1e7
Change in Qs (Source A and B) over time
Qs Population A (m^3/yr)
Qs Population B (m^3/yr)
Time (yr)
1e7

Scenario1.6

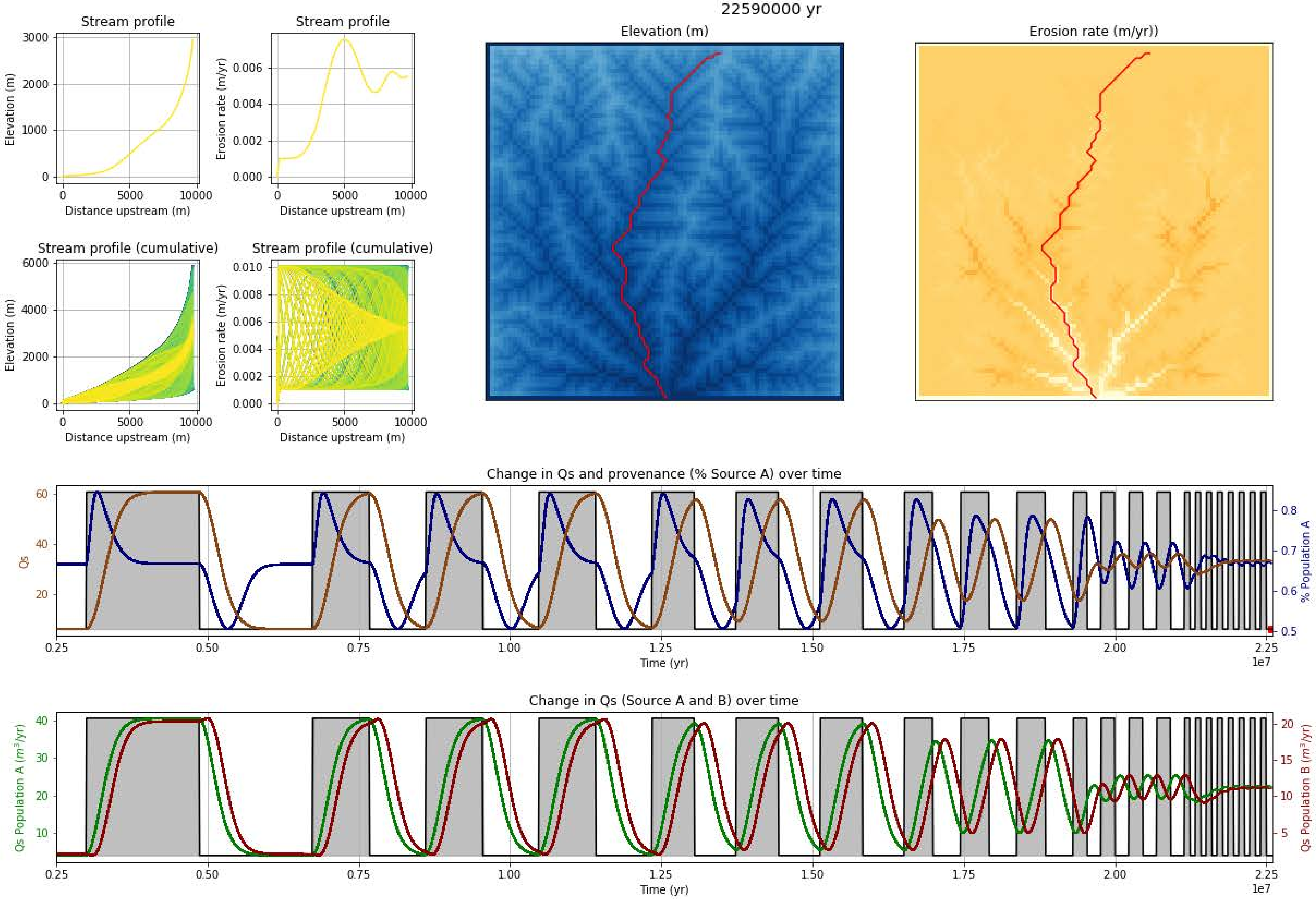

22590000 yr
Stream profile
Elevation (m)
Distance upstream (m)
Stream profile
Erosion rate (m/yr)
Distance upstream (m)
Stream profile (cumulative)
Elevation (m)
Distance upstream (m)
Stream profile (cumulative)
Erosion rate (m/yr)
Distance upstream (m)
Elevation (m)
Erosion rate (m/yr))
Change in Qs and provenance (% Source A) over time
Qs
% Population A
Time (yr)
1e7
Change in Qs (Source A and B) over time
Qs Population A (m³/yr)
Qs Population B (m³/yr)
Time (yr)
1e7

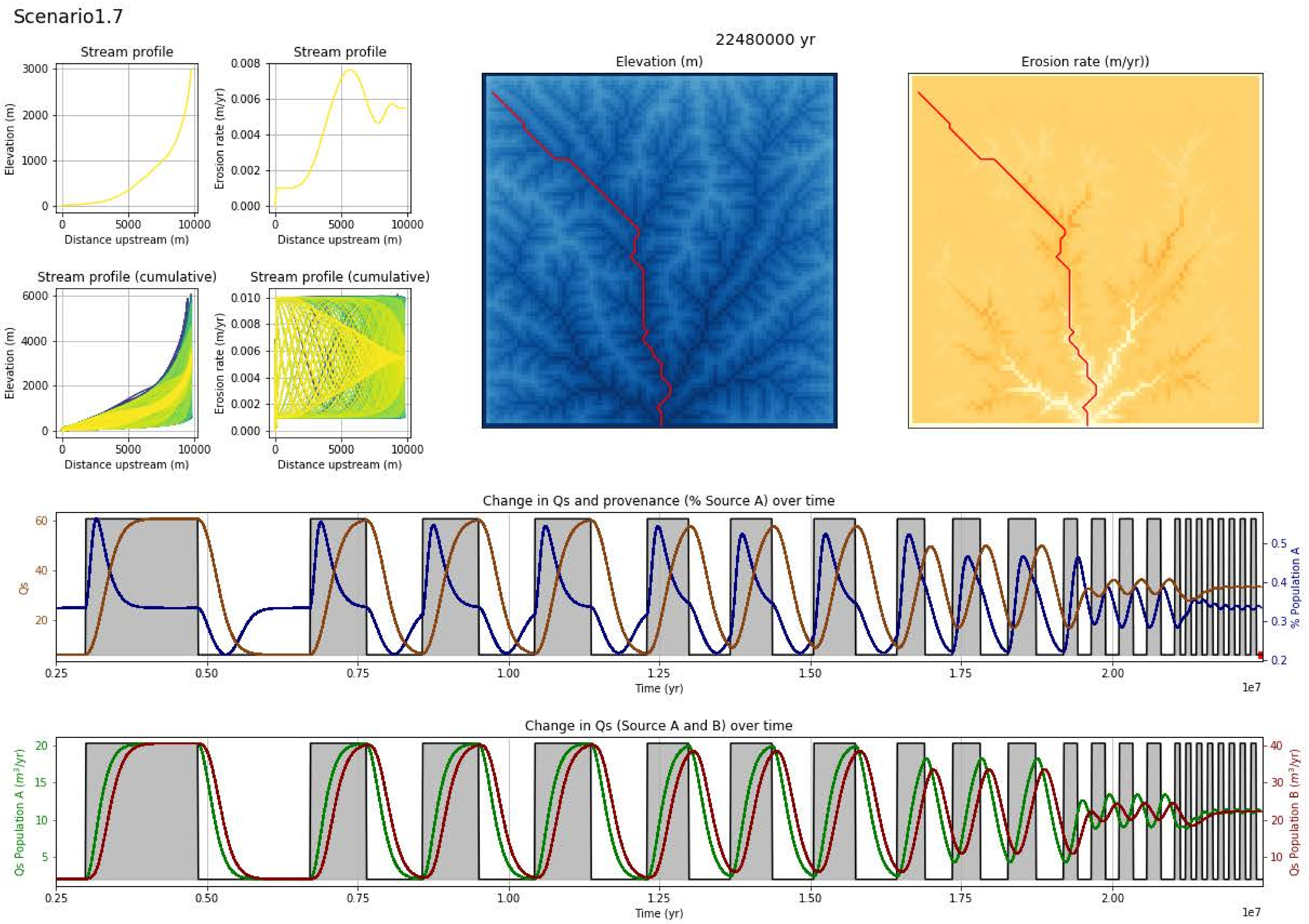

Scenario1.7
22480000 yr
Stream profile
Elevation (m)
Distance upstream (m)
Stream profile
Erosion rate (m/yr)
Distance upstream (m)
Stream profile (cumulative)
Elevation (m)
Distance upstream (m)
Stream profile (cumulative)
Erosion rate (m/yr)
Distance upstream (m)
Elevation (m)
Erosion rate (m/yr))
Change in Qs and provenance (% Source A) over time
Qs
% Population A
Time (yr)
1e7
Change in Qs (Source A and B) over time
Qs Population A (m³/yr)
Qs Population B (m³/yr)
Time (yr)
1e7

Scenario1.8

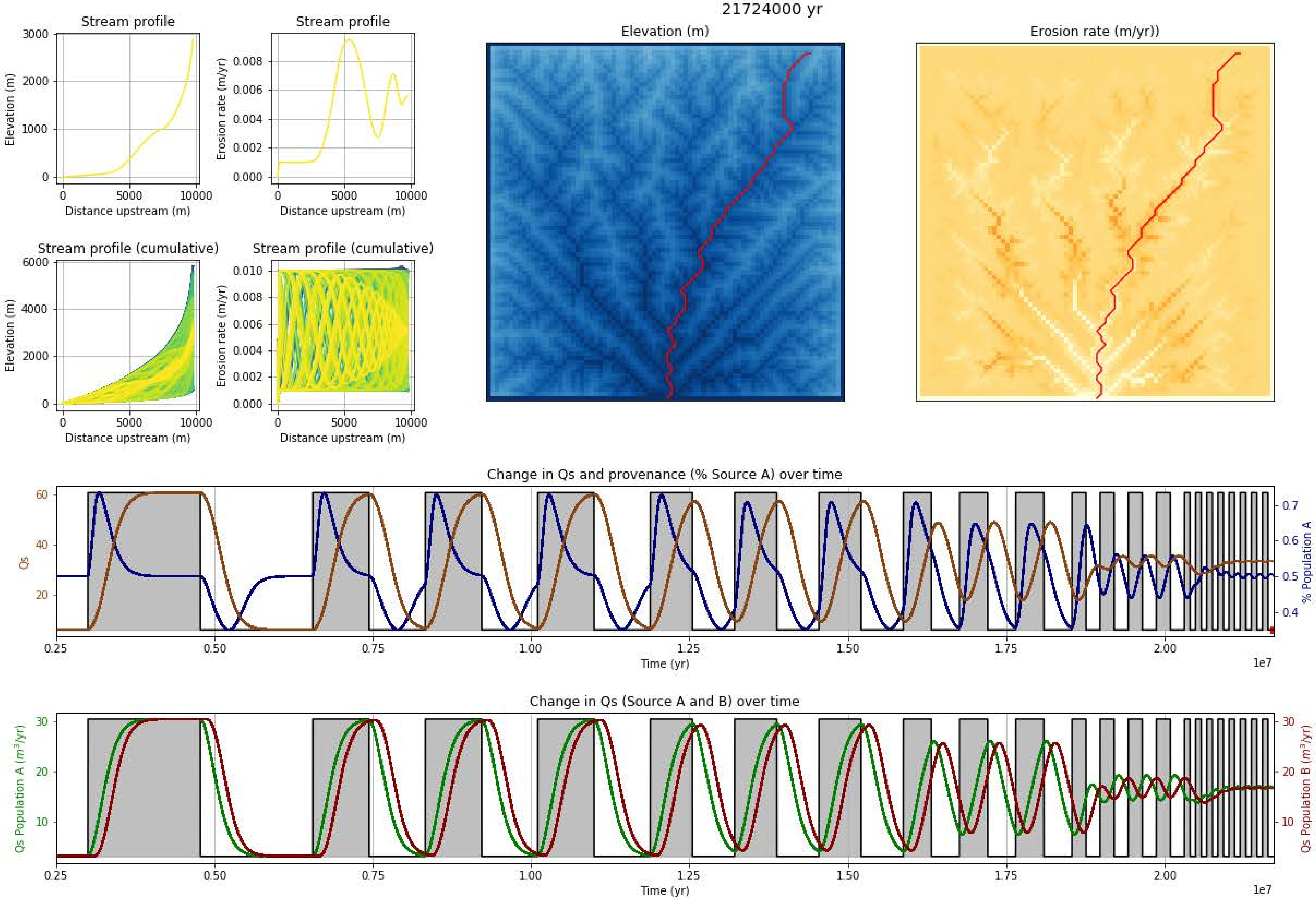
21724000 yr
Stream profile
Elevation (m)
Distance upstream (m)
Stream profile
Erosion rate (m/yr)
Distance upstream (m)
Stream profile (cumulative)
Elevation (m)
Distance upstream (m)
Stream profile (cumulative)
Erosion rate (m/yr)
Distance upstream (m)
Elevation (m)
Erosion rate (m/yr))
Change in Qs and provenance (% Source A) over time
Qs
% Population A
Time (yr)
1e7
Change in Qs (Source A and B) over time
Qs Population A (m³/yr)
Qs Population B (m³/yr)
Time (yr)
1e7

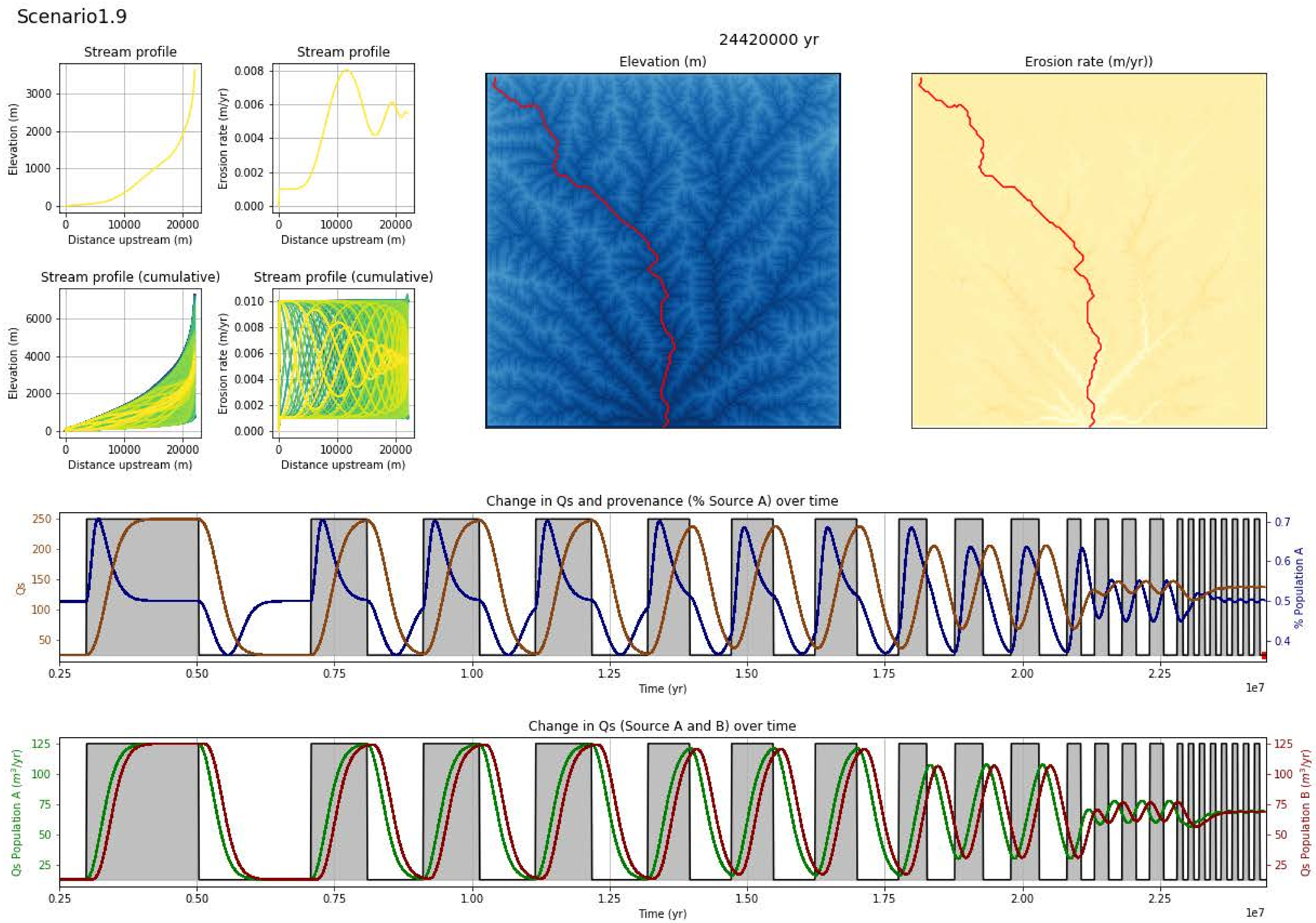

Scenario1.9
Stream profile
Elevation (m)
Distance upstream (m)
Stream profile
Erosion rate (m/yr)
Distance upstream (m)
Stream profile (cumulative)
Elevation (m)
Distance upstream (m)
Stream profile (cumulative)
Erosion rate (m/yr)
Distance upstream (m)
24420000 yr
Elevation (m)
Erosion rate (m/yr))
Change in Qs and provenance (% Source A) over time
Qs
% Population A
Time (yr)
1e7
Change in Qs (Source A and B) over time
Qs Population A (m^3/yr)
Qs Population B (m^3/yr)
Time (yr)
1e7

Scenario1.10

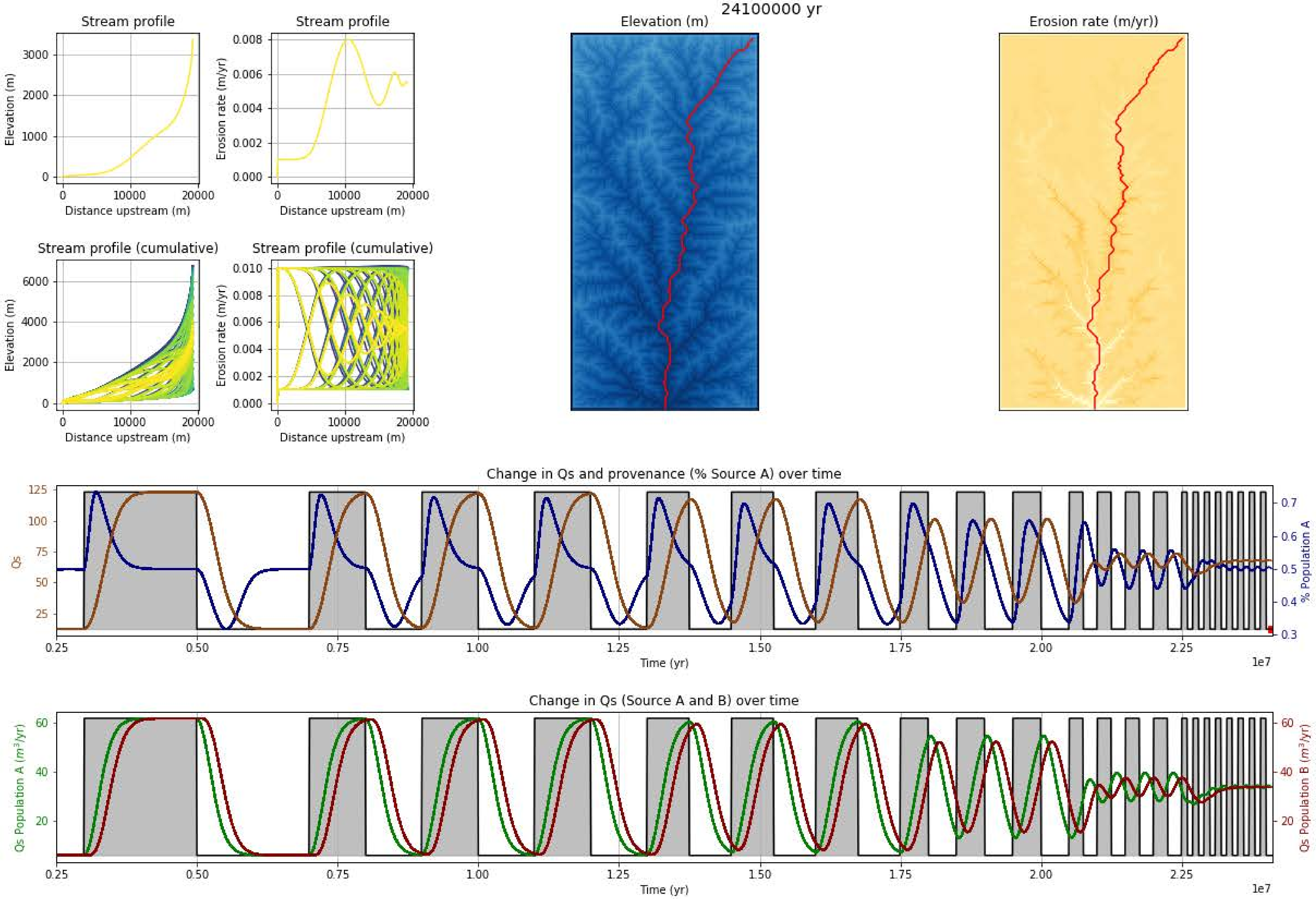

Scenario1.11

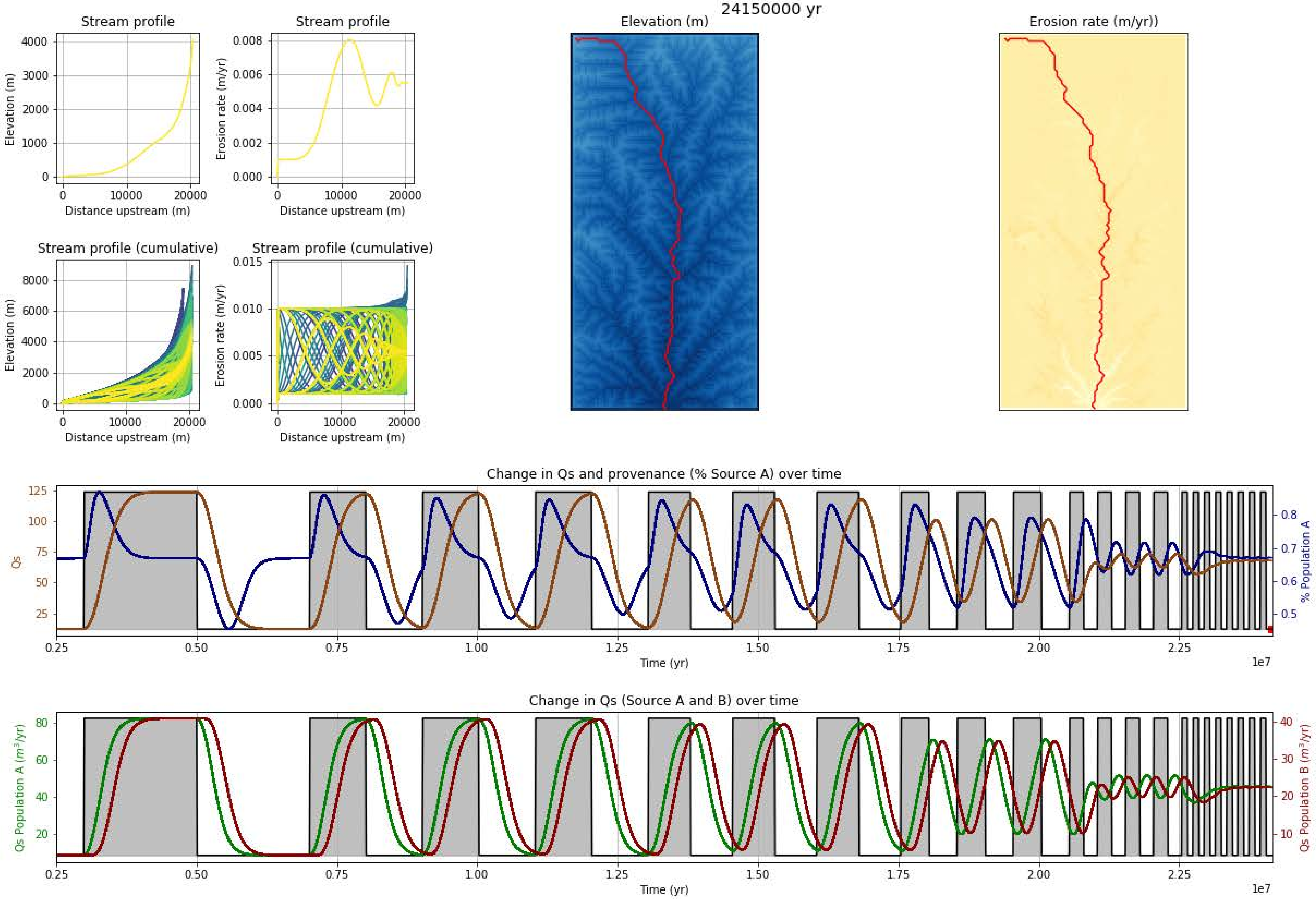

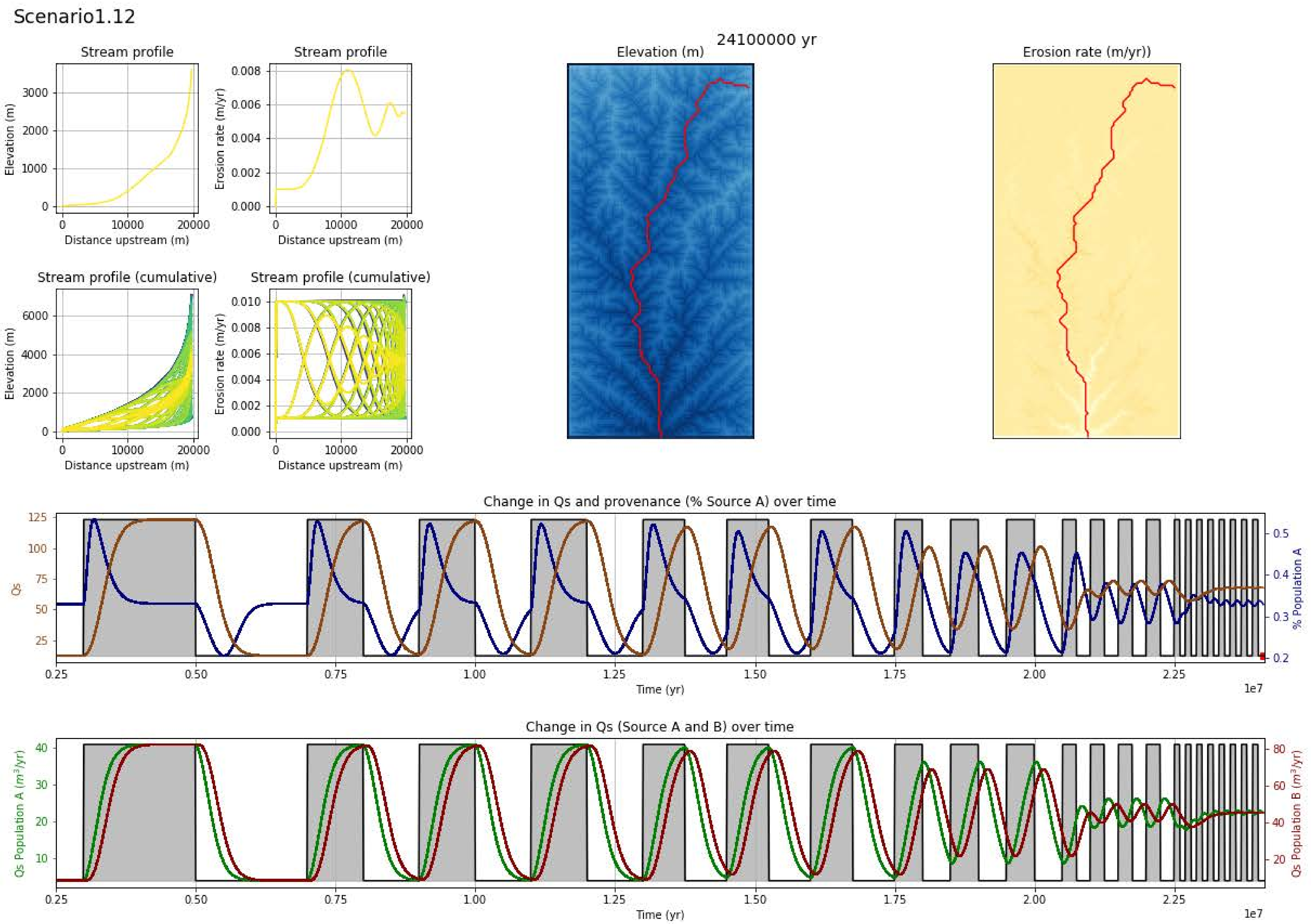

Scenario1.12
24100000 yr
Stream profile
Elevation (m)
Distance upstream (m)
Stream profile
Erosion rate (m/yr)
Distance upstream (m)
Stream profile (cumulative)
Elevation (m)
Distance upstream (m)
Stream profile (cumulative)
Erosion rate (m/yr)
Distance upstream (m)
Elevation (m)
Erosion rate (m/yr))
Change in Qs and provenance (% Source A) over time
Qs
% Population A
Time (yr)
1e7
Change in Qs (Source A and B) over time
Qs Population A (m³/yr)
Qs Population B (m³/yr)
Time (yr)
1e7

Scenario1.13

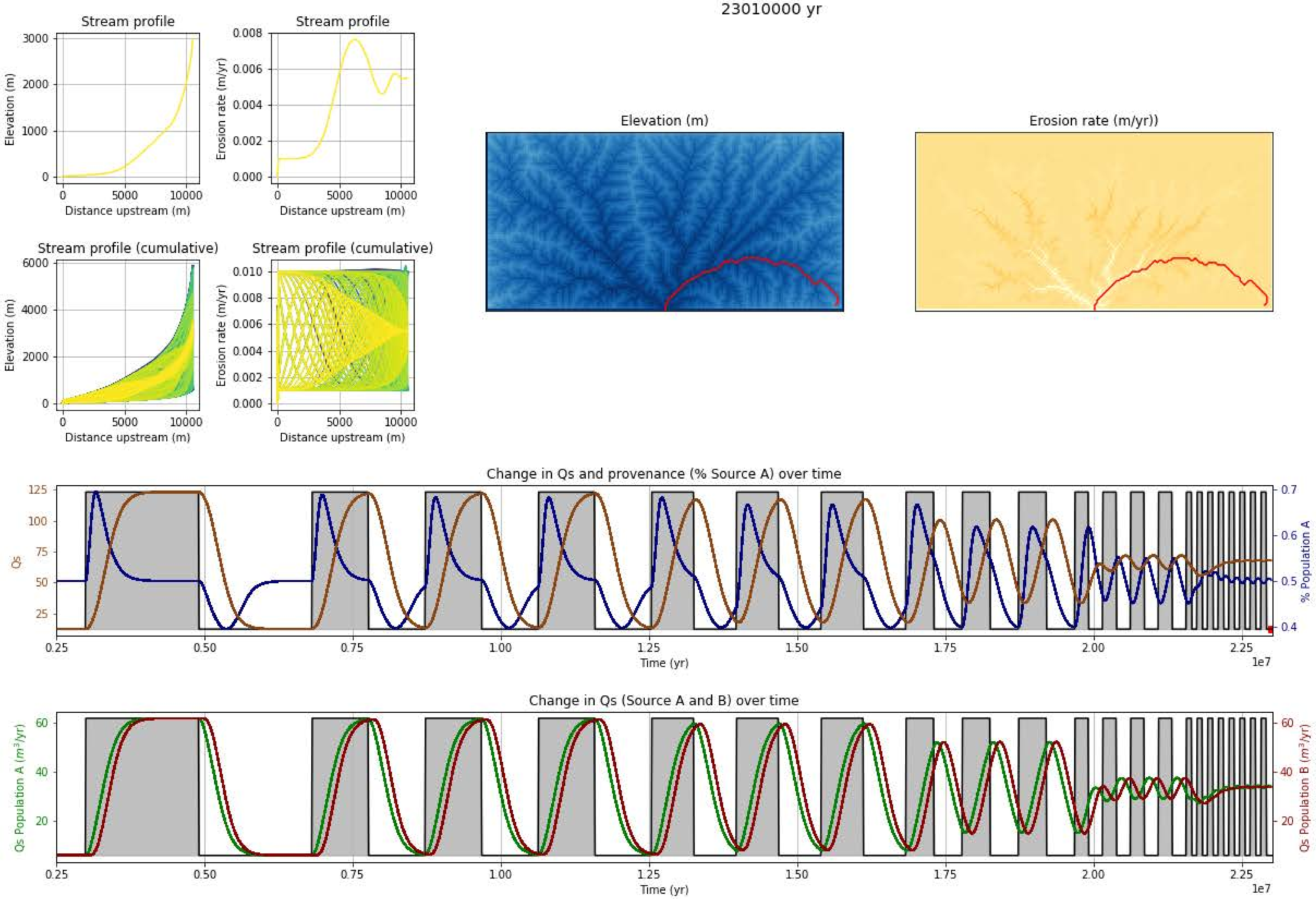

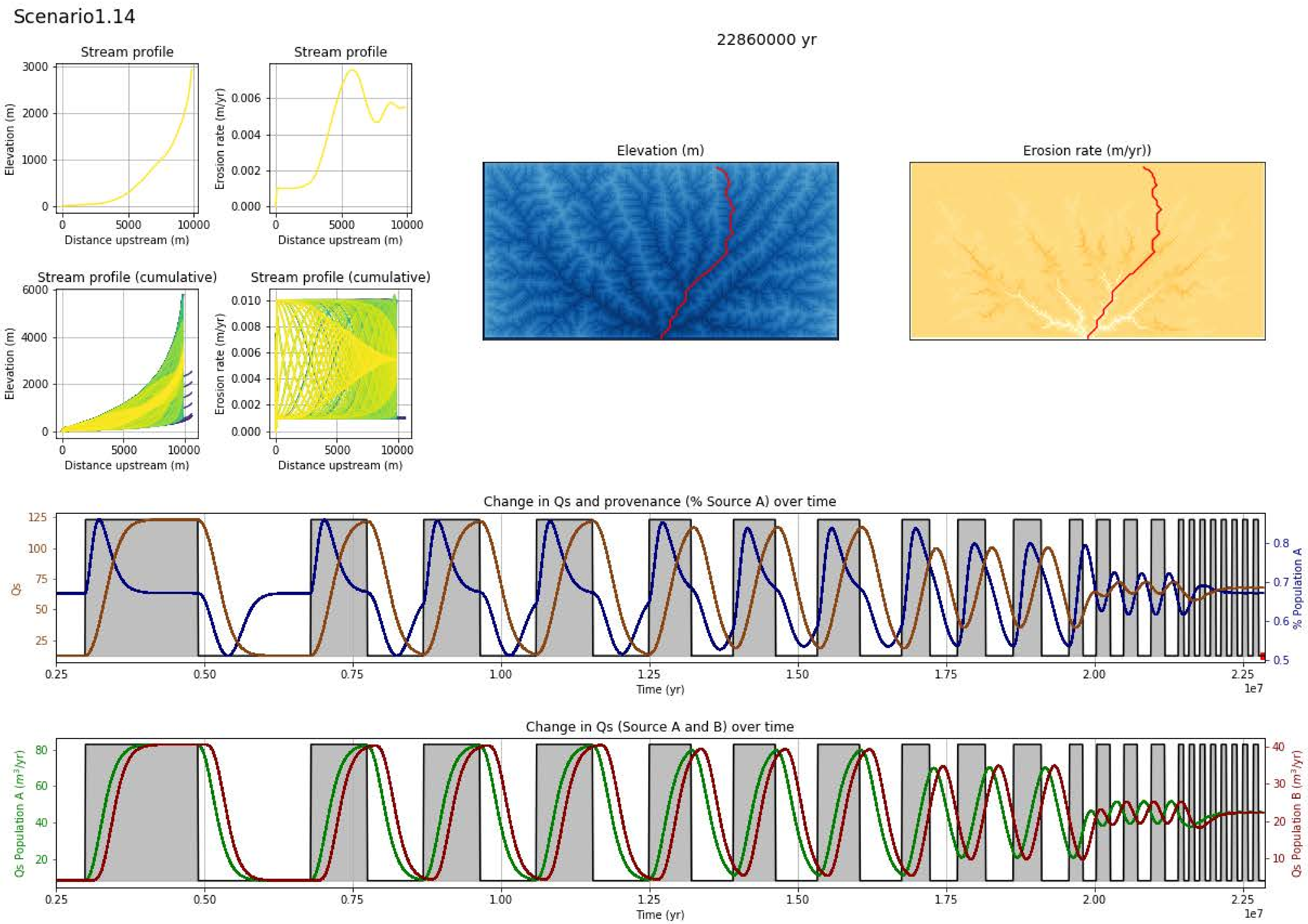

Scenario1.14
22860000 yr
Stream profile
Elevation (m)
Distance upstream (m)
Stream profile
Erosion rate (m/yr)
Distance upstream (m)
Stream profile (cumulative)
Elevation (m)
Distance upstream (m)
Stream profile (cumulative)
Erosion rate (m/yr)
Distance upstream (m)
Elevation (m)
Erosion rate (m/yr))
Change in Qs and provenance (% Source A) over time
Qs
% Population A
Time (yr)
1e7
Change in Qs (Source A and B) over time
Qs Population A (m³/yr)
Qs Population B (m³/yr)
Time (yr)
1e7

Scenario1.15

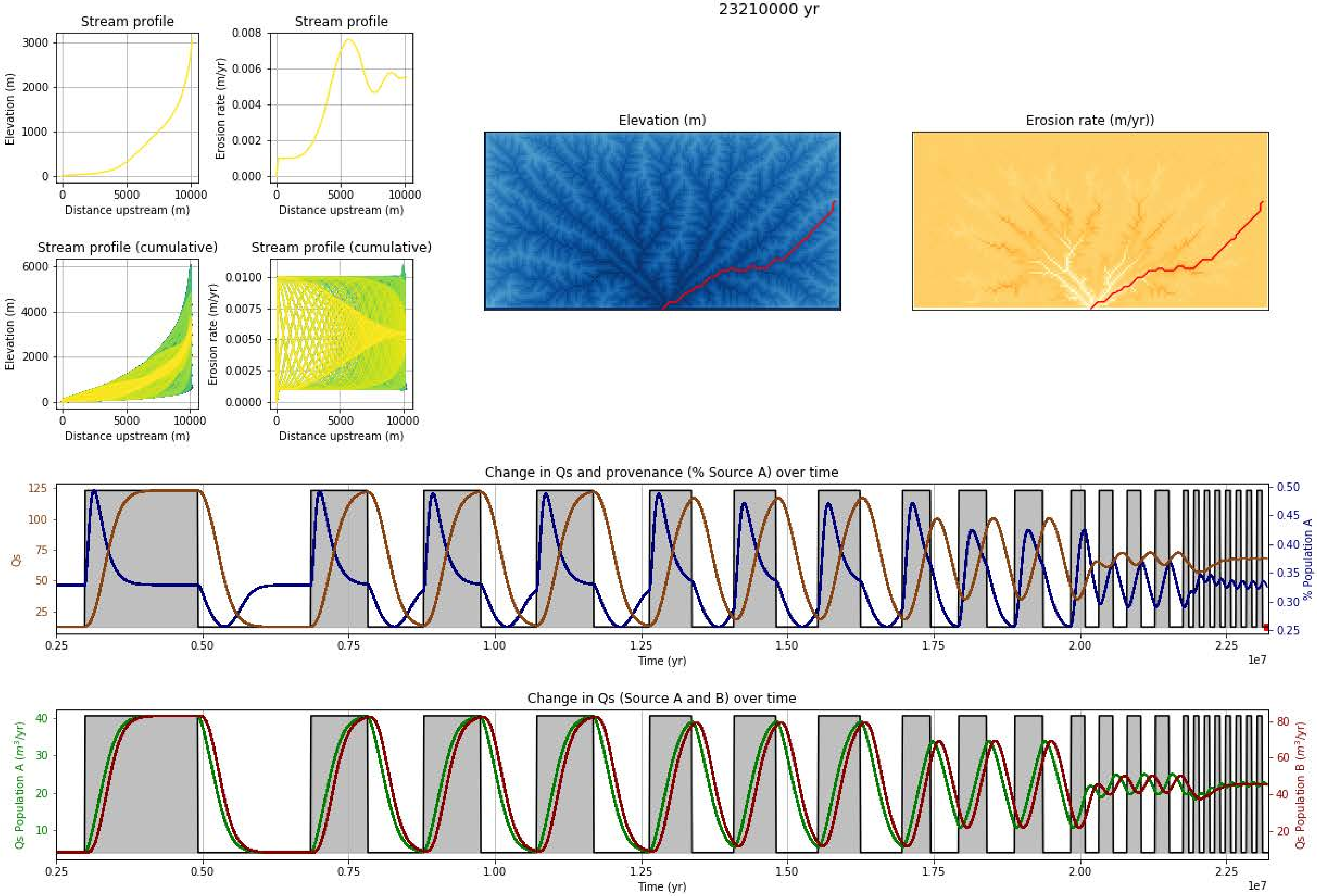

Scenario2.1

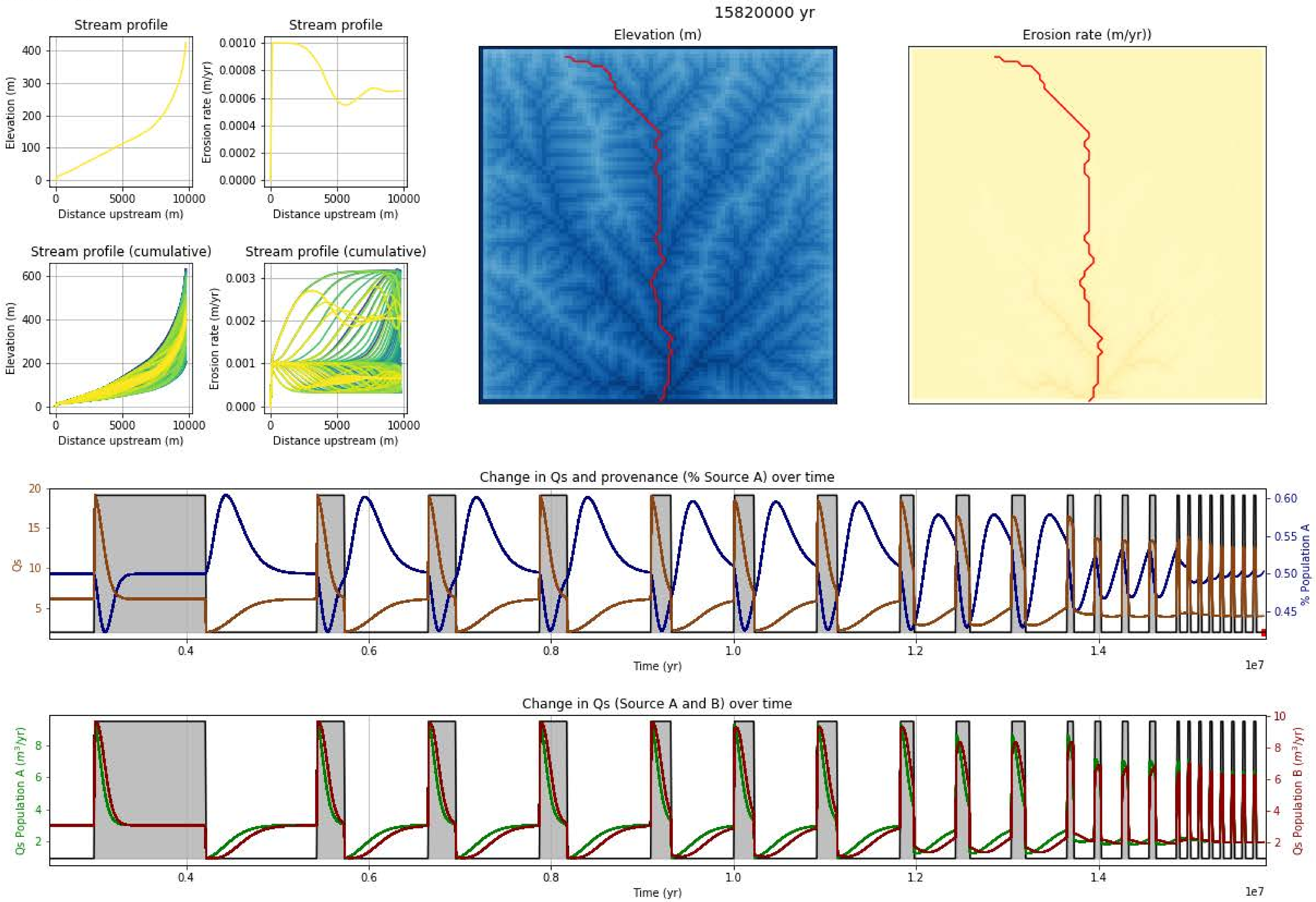
15820000 yr
Stream profile
Elevation (m)
Distance upstream (m)
Stream profile
Erosion rate (m/yr)
Distance upstream (m)
Stream profile (cumulative)
Elevation (m)
Distance upstream (m)
Stream profile (cumulative)
Erosion rate (m/yr)
Distance upstream (m)
Elevation (m)
Erosion rate (m/yr))
Change in Qs and provenance (% Source A) over time
Qs
% Population A
Time (yr)
1e7
Change in Qs (Source A and B) over time
Qs Population A (m³/yr)
Qs Population B (m³/yr)
Time (yr)
1e7

Scenario2.2

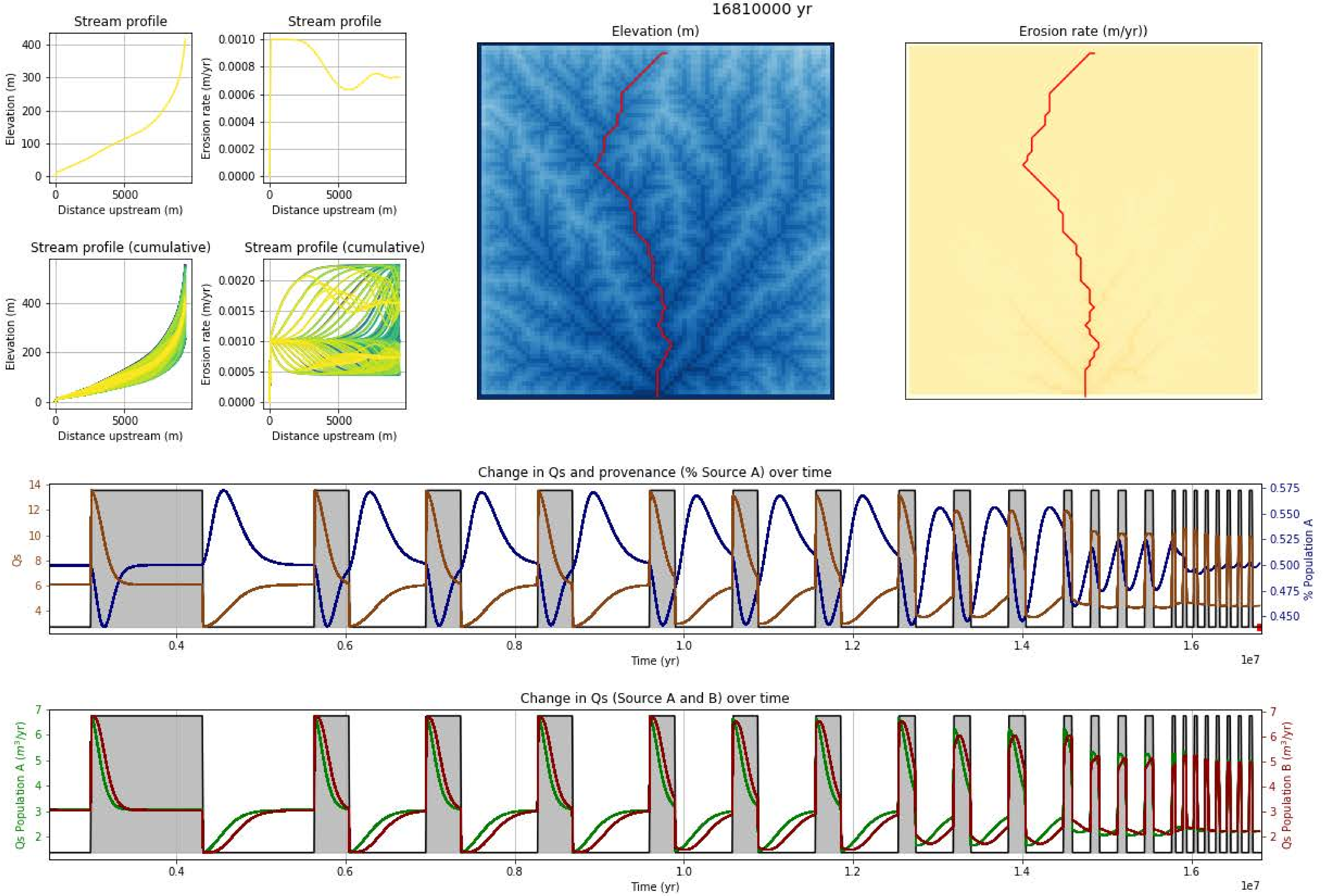

16810000 yr
Stream profile
Elevation (m)
Distance upstream (m)
Erosion rate (m/yr)
Stream profile (cumulative)
Elevation (m)
Erosion rate (m/yr))
Change in Qs and provenance (% Source A) over time
Qs
% Population A
Time (yr)
1e7
Change in Qs (Source A and B) over time
Qs Population A (m^3/yr)
Qs Population B (m^3/yr)

Scenario2.3

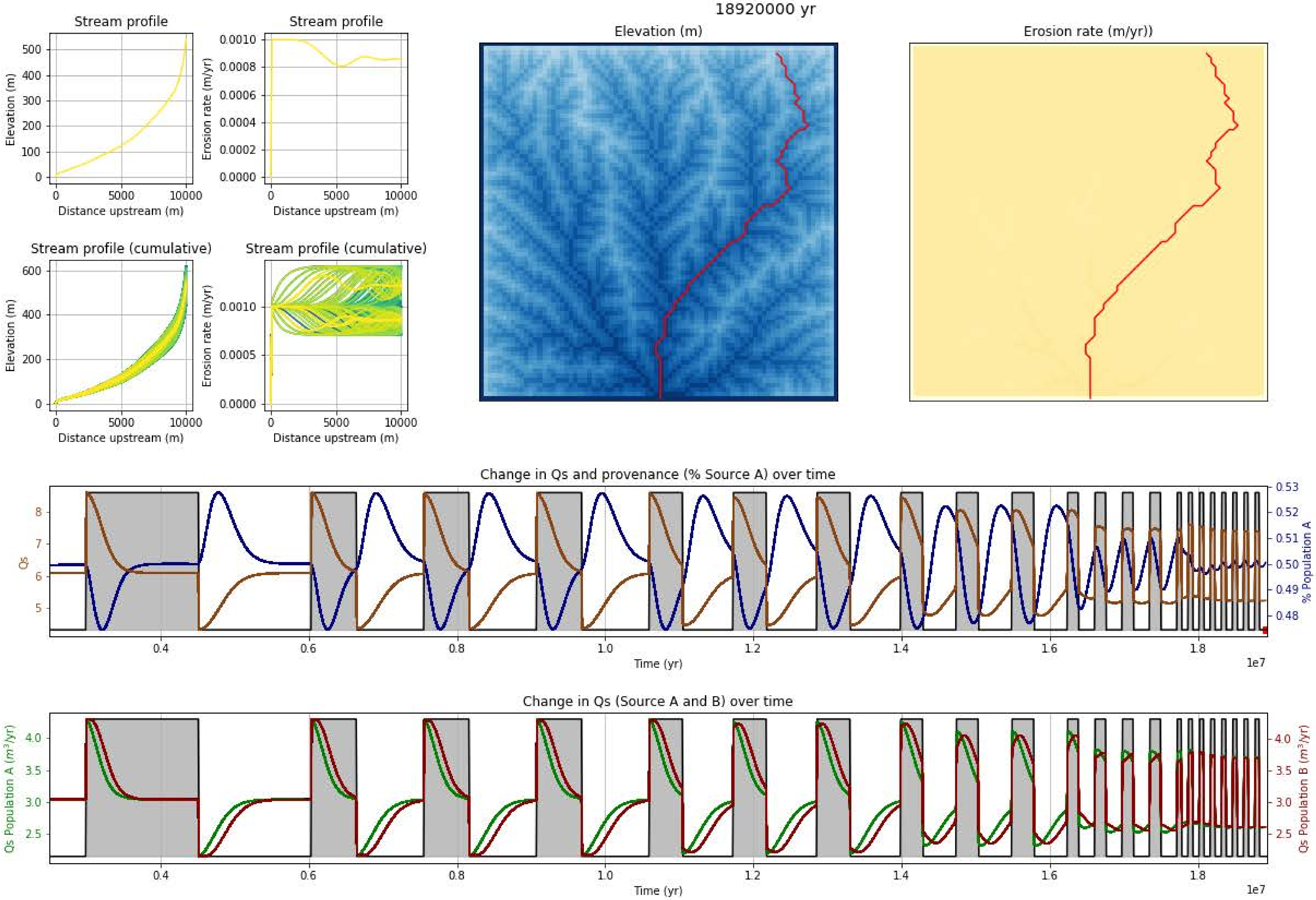

18920000 yr
Stream profile
Elevation (m)
Distance upstream (m)
Stream profile
Erosion rate (m/yr)
Distance upstream (m)
Stream profile (cumulative)
Elevation (m)
Distance upstream (m)
Stream profile (cumulative)
Erosion rate (m/yr)
Distance upstream (m)
Elevation (m)
Erosion rate (m/yr))
Change in Qs and provenance (% Source A) over time
Qs
% Population A
Time (yr)
1e7
Change in Qs (Source A and B) over time
Qs Population A (m^3/yr)
Qs Population B (m^3/yr)
Time (yr)
1e7

Scenario2.4

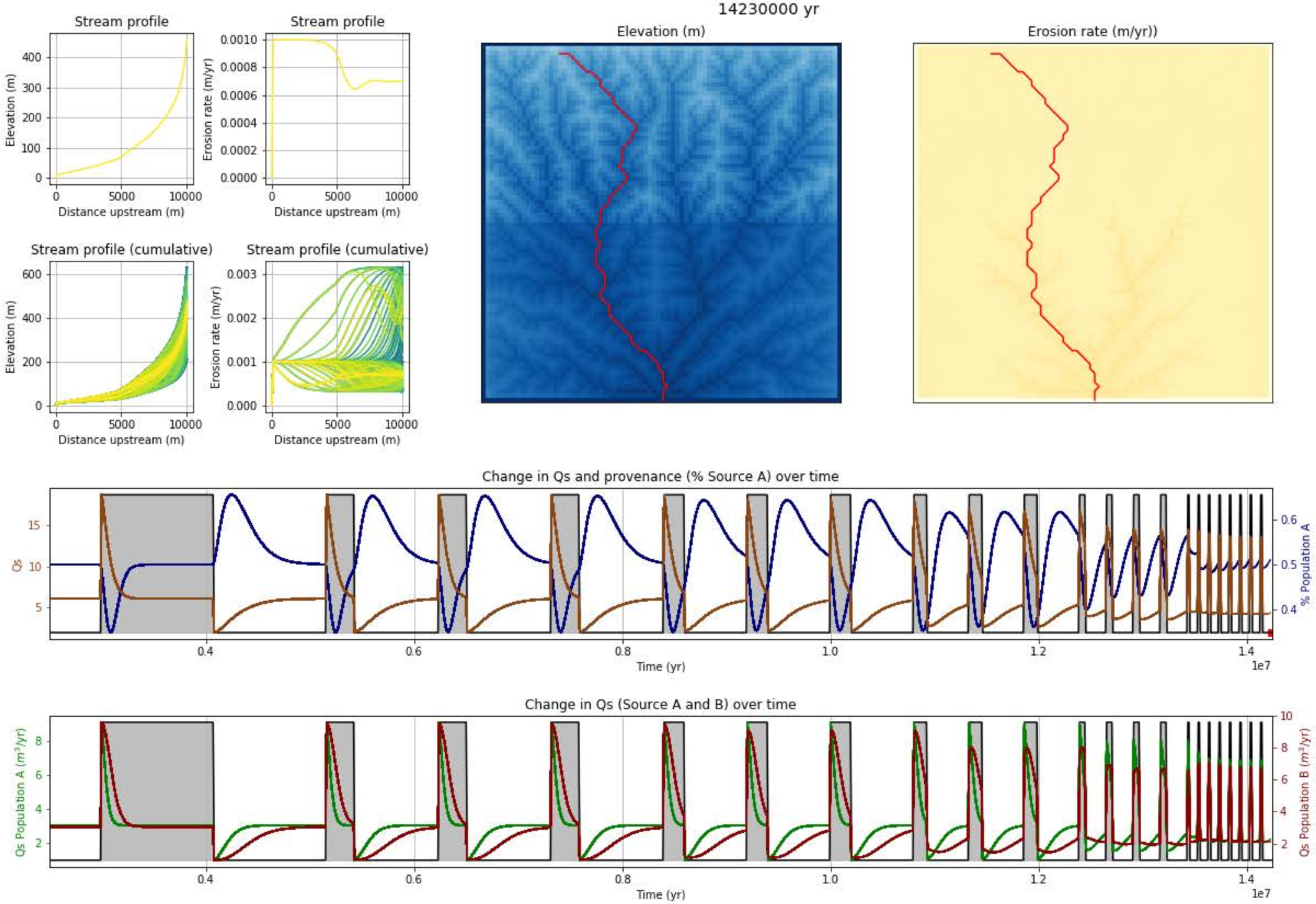

14230000 yr
Stream profile
Elevation (m)
Distance upstream (m)
Stream profile
Erosion rate (m/yr)
Distance upstream (m)
Stream profile (cumulative)
Elevation (m)
Distance upstream (m)
Stream profile (cumulative)
Erosion rate (m/yr)
Distance upstream (m)
Elevation (m)
Erosion rate (m/yr))
Change in Qs and provenance (% Source A) over time
Qs
% Population A
Time (yr)
1e7
Change in Qs (Source A and B) over time
Qs Population A (m³/yr)
Qs Population B (m³/yr)
Time (yr)
1e7

Scenario2.5

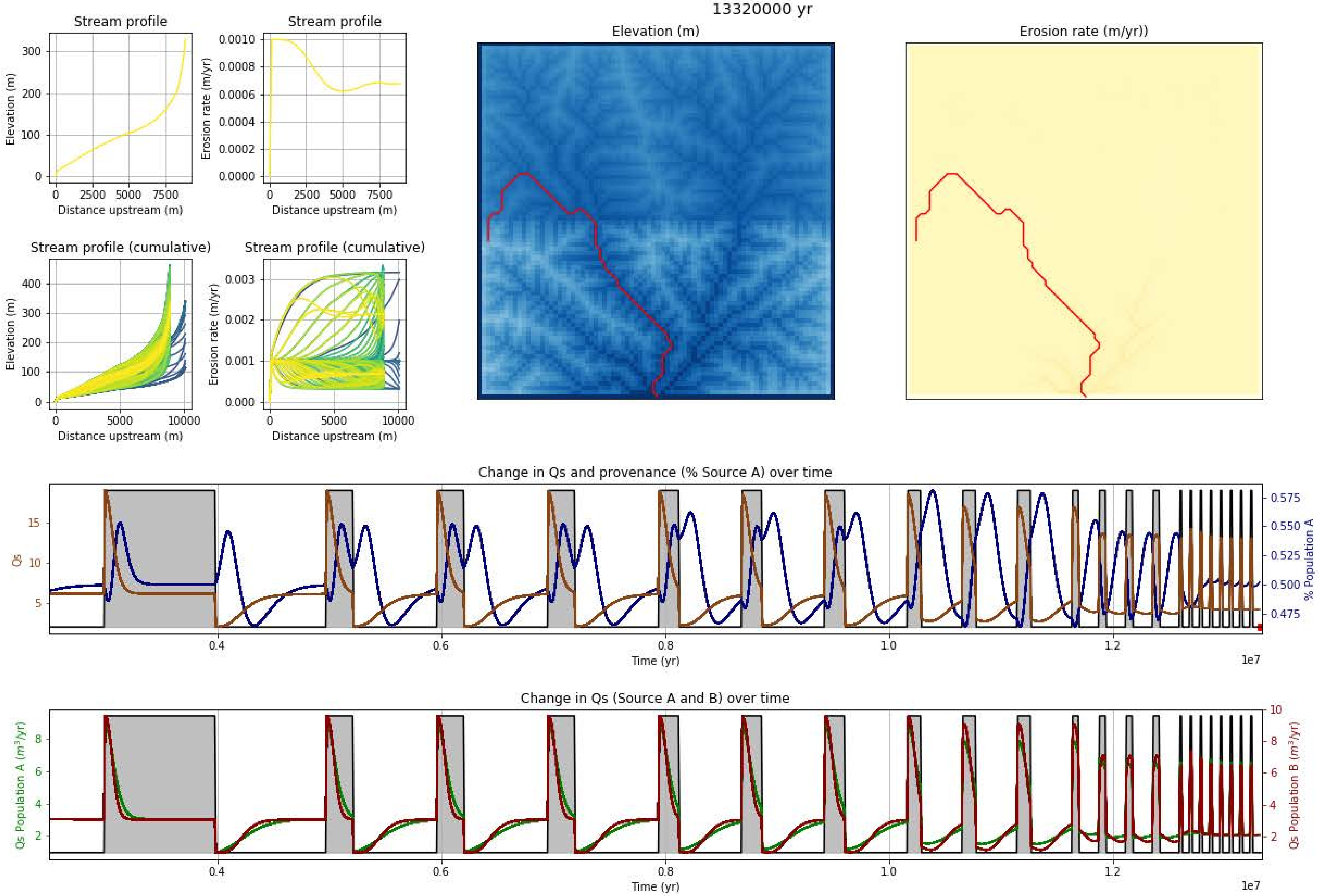

13320000 yr
Stream profile
Elevation (m)
Distance upstream (m)
Stream profile
Erosion rate (m/yr)
Distance upstream (m)
Elevation (m)
Erosion rate (m/yr))
Stream profile (cumulative)
Elevation (m)
Distance upstream (m)
Stream profile (cumulative)
Erosion rate (m/yr)
Distance upstream (m)
Change in Qs and provenance (% Source A) over time
Qs
% Population A
Time (yr)
1e7
Change in Qs (Source A and B) over time
Qs Population A (m³/yr)
Qs Population B (m³/yr)
Time (yr)
1e7

Scenario2.6

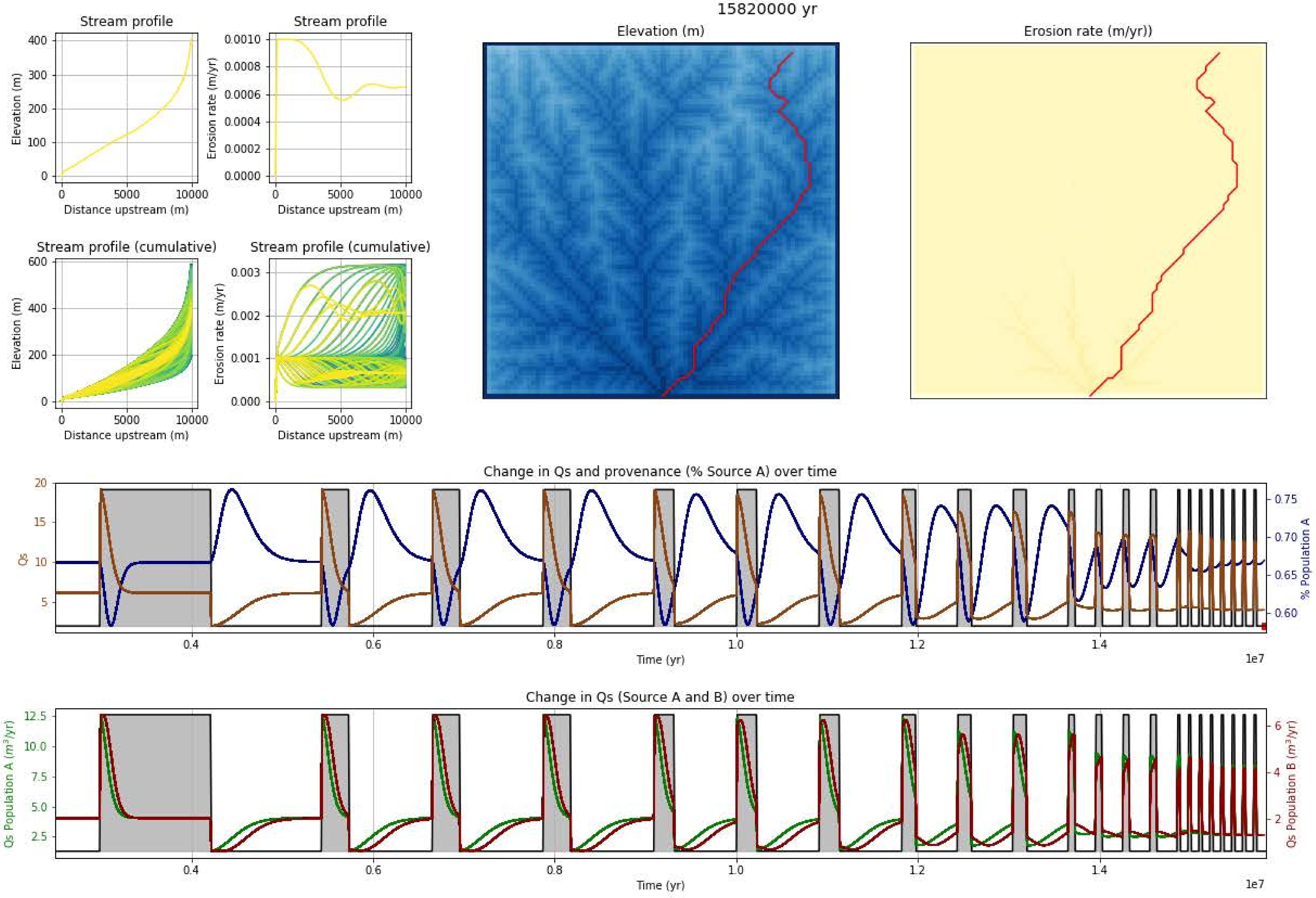

15820000 yr
Stream profile
Elevation (m)
Distance upstream (m)
Stream profile
Erosion rate (m/yr)
Distance upstream (m)
Stream profile (cumulative)
Elevation (m)
Distance upstream (m)
Stream profile (cumulative)
Erosion rate (m/yr)
Distance upstream (m)
Elevation (m)
Erosion rate (m/yr))
Change in Qs and provenance (% Source A) over time
Qs
% Population A
Time (yr)
1e7
Change in Qs (Source A and B) over time
Qs Population A (m^3/yr)
Qs Population B (m^3/yr)
Time (yr)
1e7

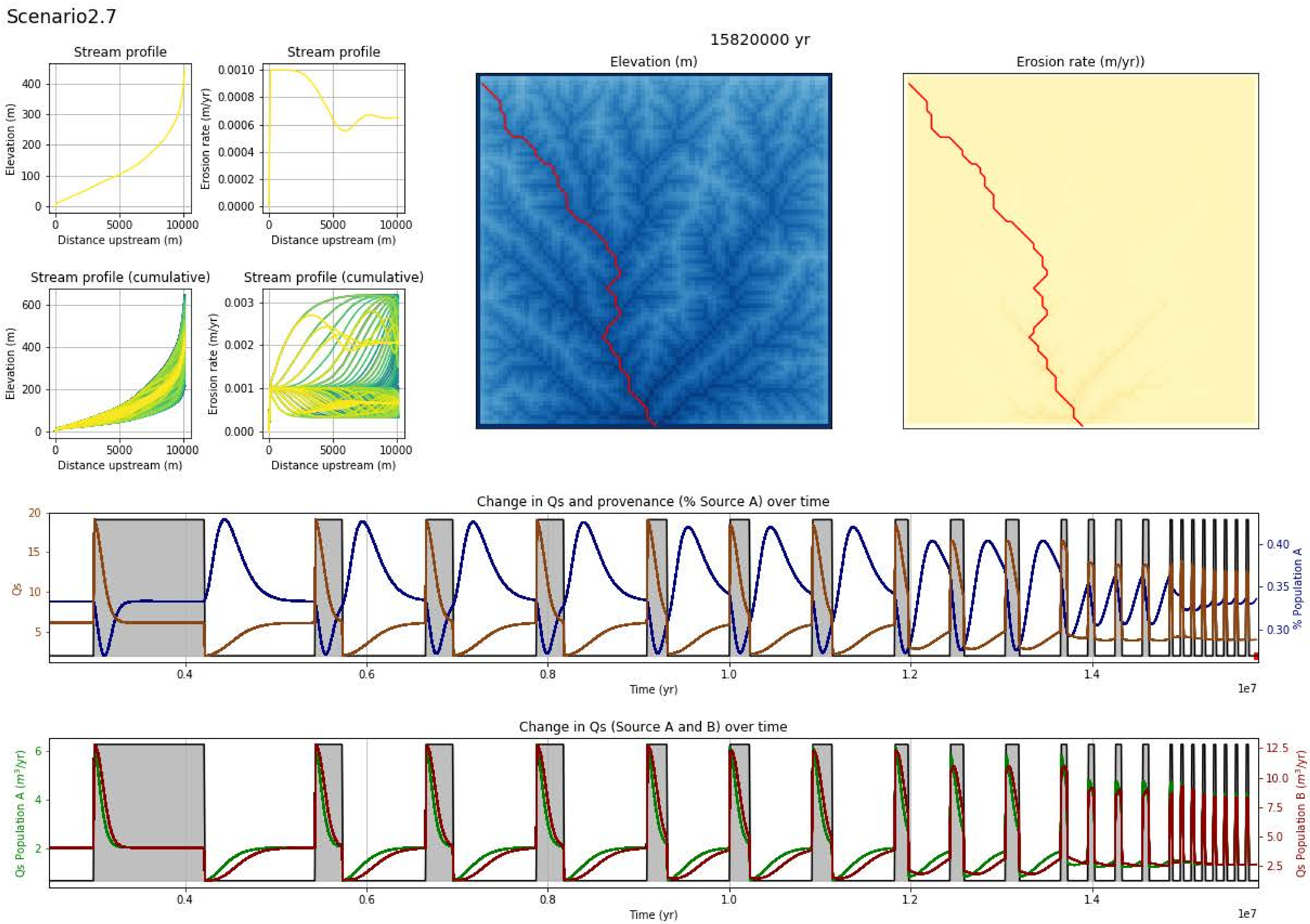

Scenario2.7
15820000 yr
Stream profile
Elevation (m)
Distance upstream (m)
Stream profile
Erosion rate (m/yr)
Distance upstream (m)
Stream profile (cumulative)
Elevation (m)
Distance upstream (m)
Stream profile (cumulative)
Erosion rate (m/yr)
Distance upstream (m)
Elevation (m)
Erosion rate (m/yr))
Change in Qs and provenance (% Source A) over time
Qs
% Population A
Time (yr)
1e7
Change in Qs (Source A and B) over time
Qs Population A (m^3/yr)
Qs Population B (m^3/yr)
Time (yr)
1e7

Scenario2.8

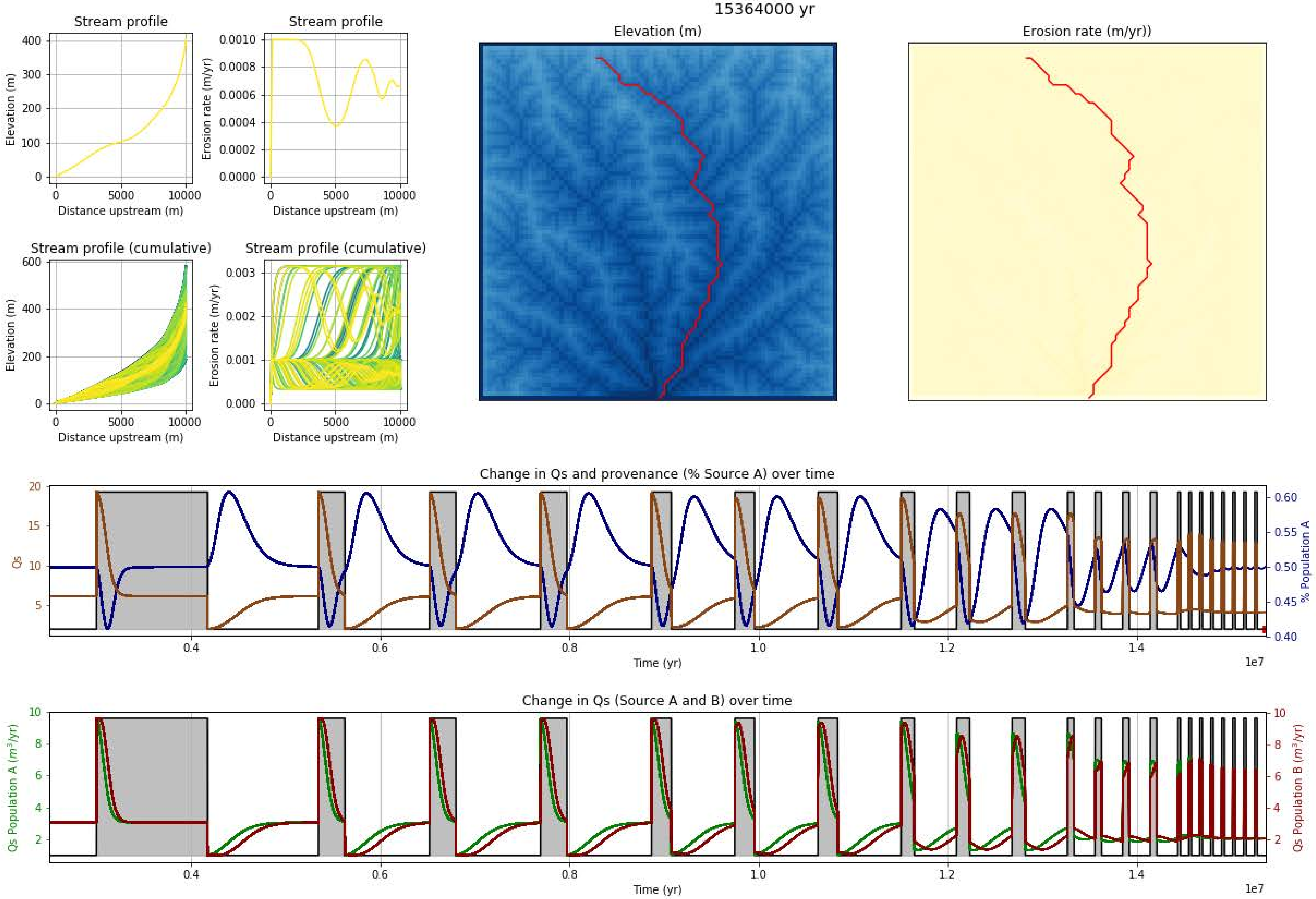

15364000 yr
Stream profile
Elevation (m)
Distance upstream (m)
Stream profile
Erosion rate (m/yr)
Distance upstream (m)
Stream profile (cumulative)
Elevation (m)
Distance upstream (m)
Stream profile (cumulative)
Erosion rate (m/yr)
Distance upstream (m)
Elevation (m)
Erosion rate (m/yr))
Change in Qs and provenance (% Source A) over time
Qs
% Population A
Time (yr)
1e7
Change in Qs (Source A and B) over time
Qs Population A (m³/yr)
Qs Population B (m³/yr)
Time (yr)
1e7

Scenario2.9

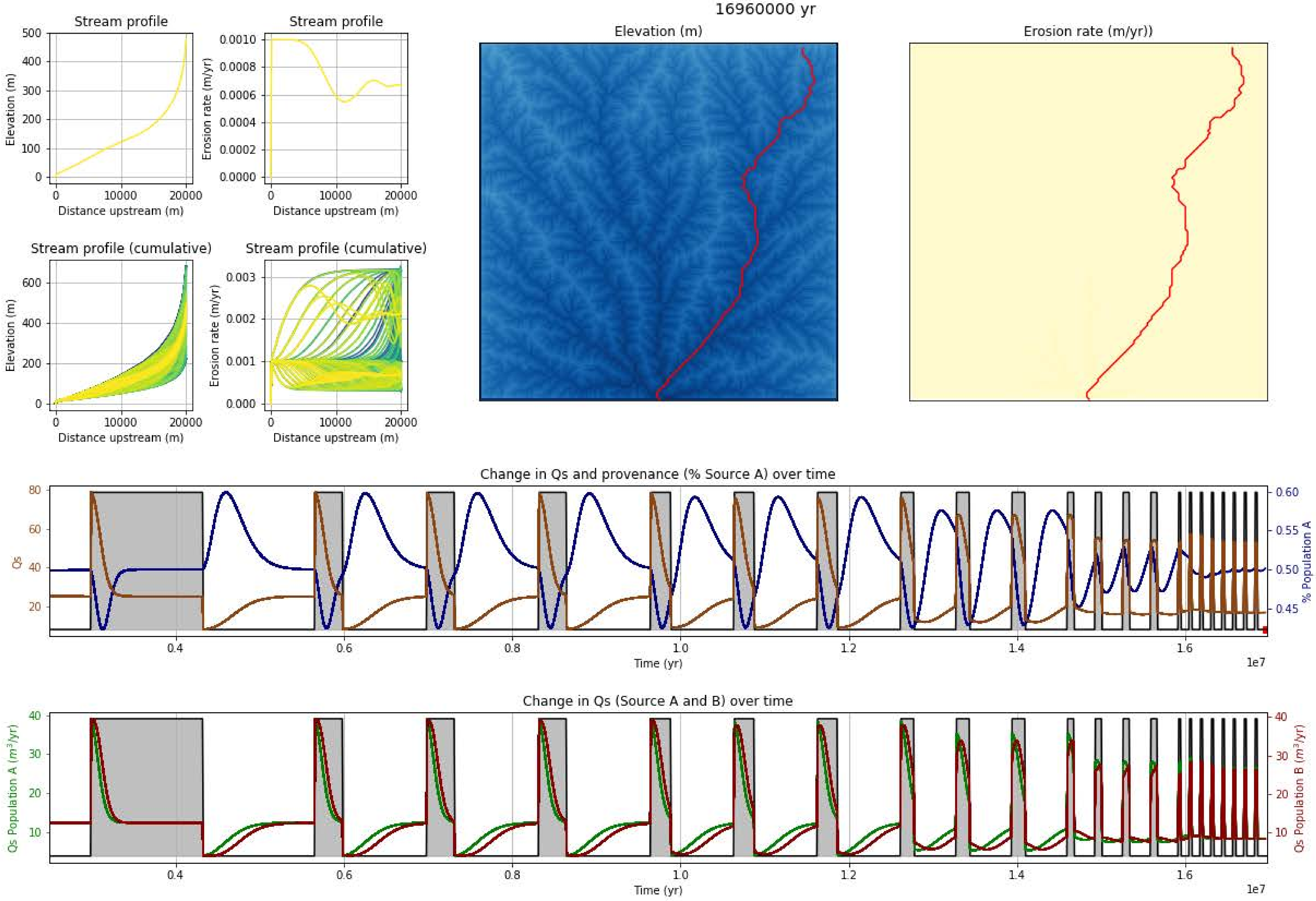

16960000 yr
Stream profile
Elevation (m)
Distance upstream (m)
Stream profile
Erosion rate (m/yr)
Distance upstream (m)
Stream profile (cumulative)
Elevation (m)
Distance upstream (m)
Stream profile (cumulative)
Erosion rate (m/yr)
Distance upstream (m)
Elevation (m)
Erosion rate (m/yr))
Change in Qs and provenance (% Source A) over time
Qs
% Population A
Time (yr)
1e7
Change in Qs (Source A and B) over time
Qs Population A (m³/yr)
Qs Population B (m³/yr)
Time (yr)
1e7

Scenario2.10

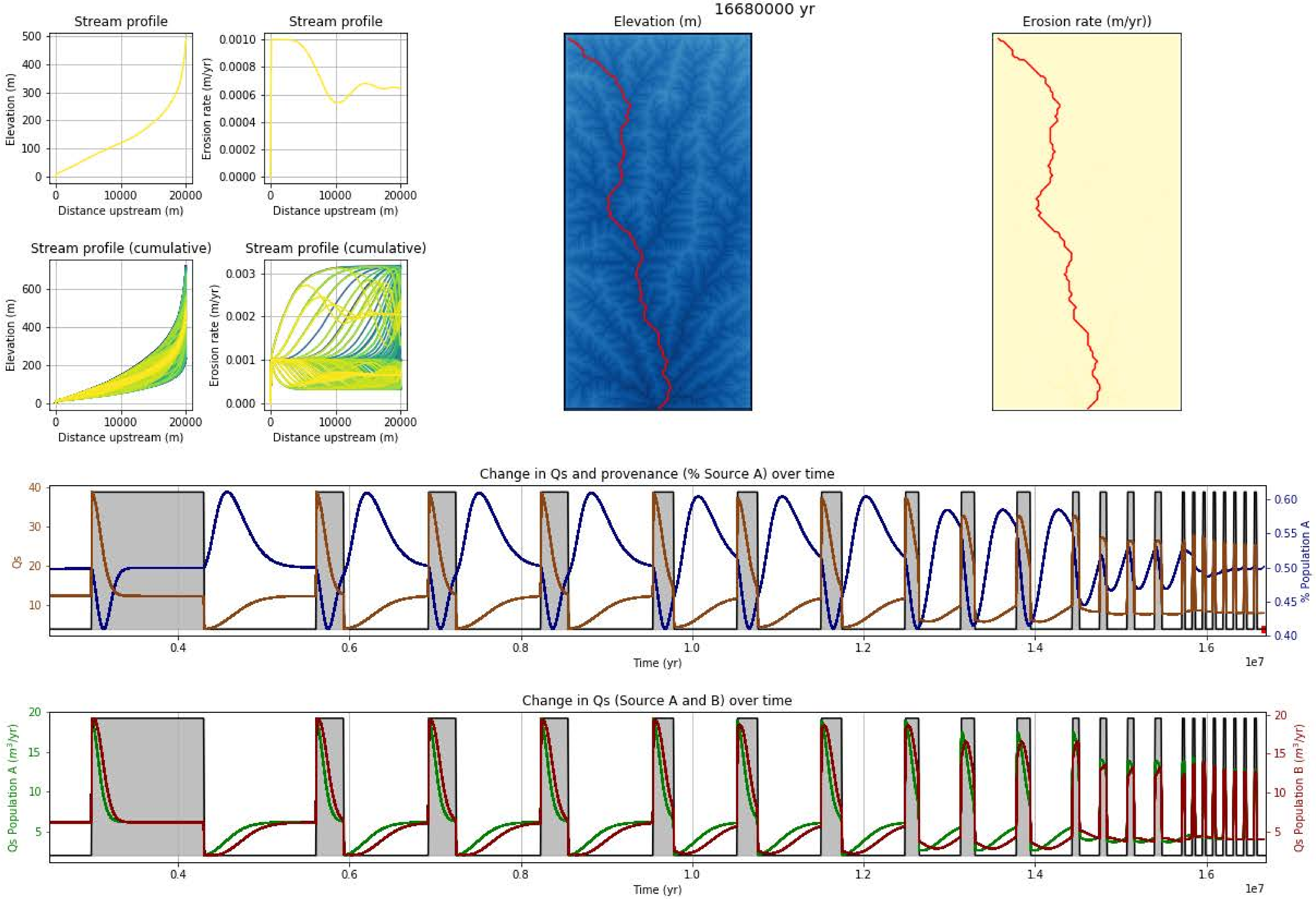

Scenario2.11

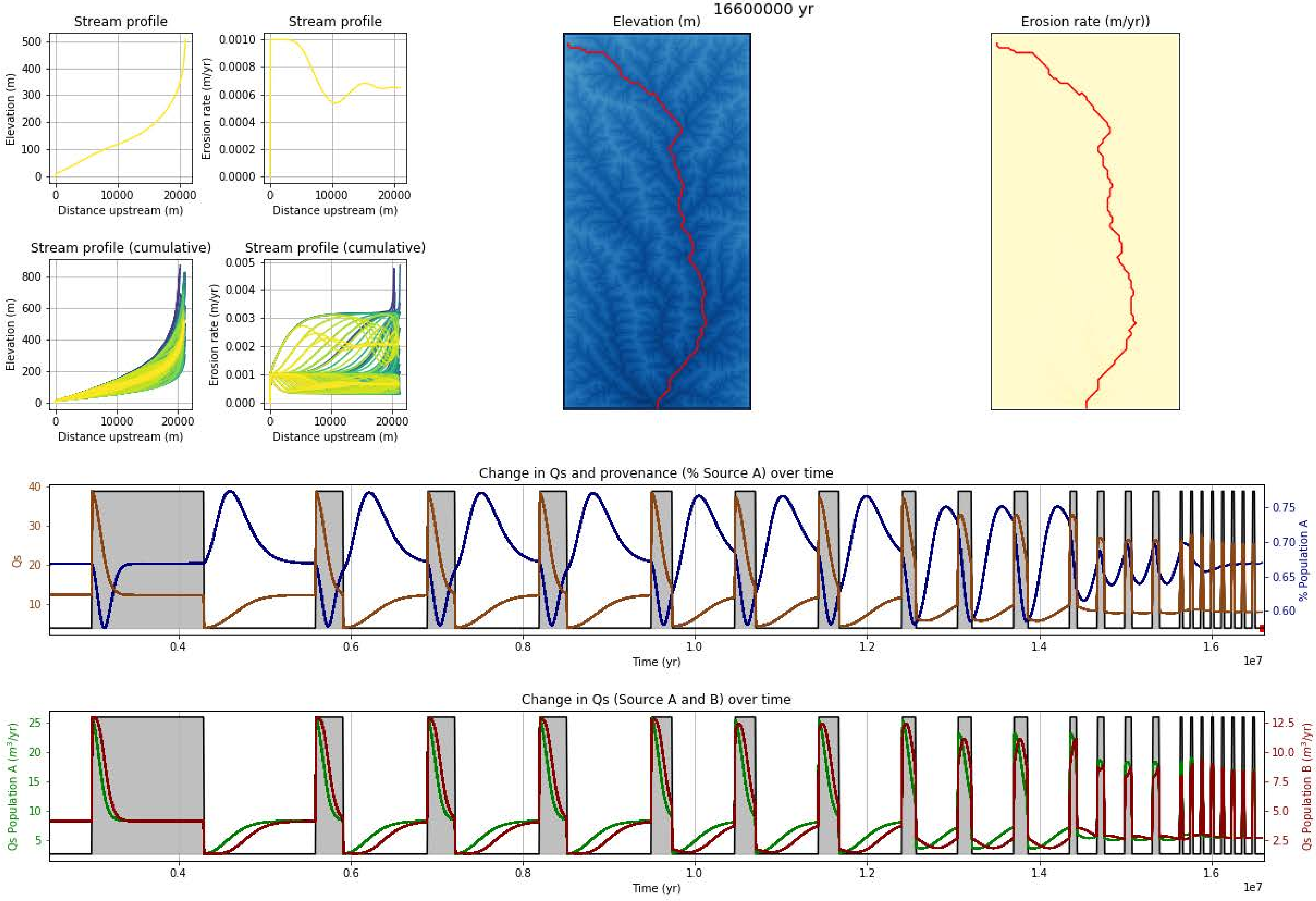

Scenario2.12

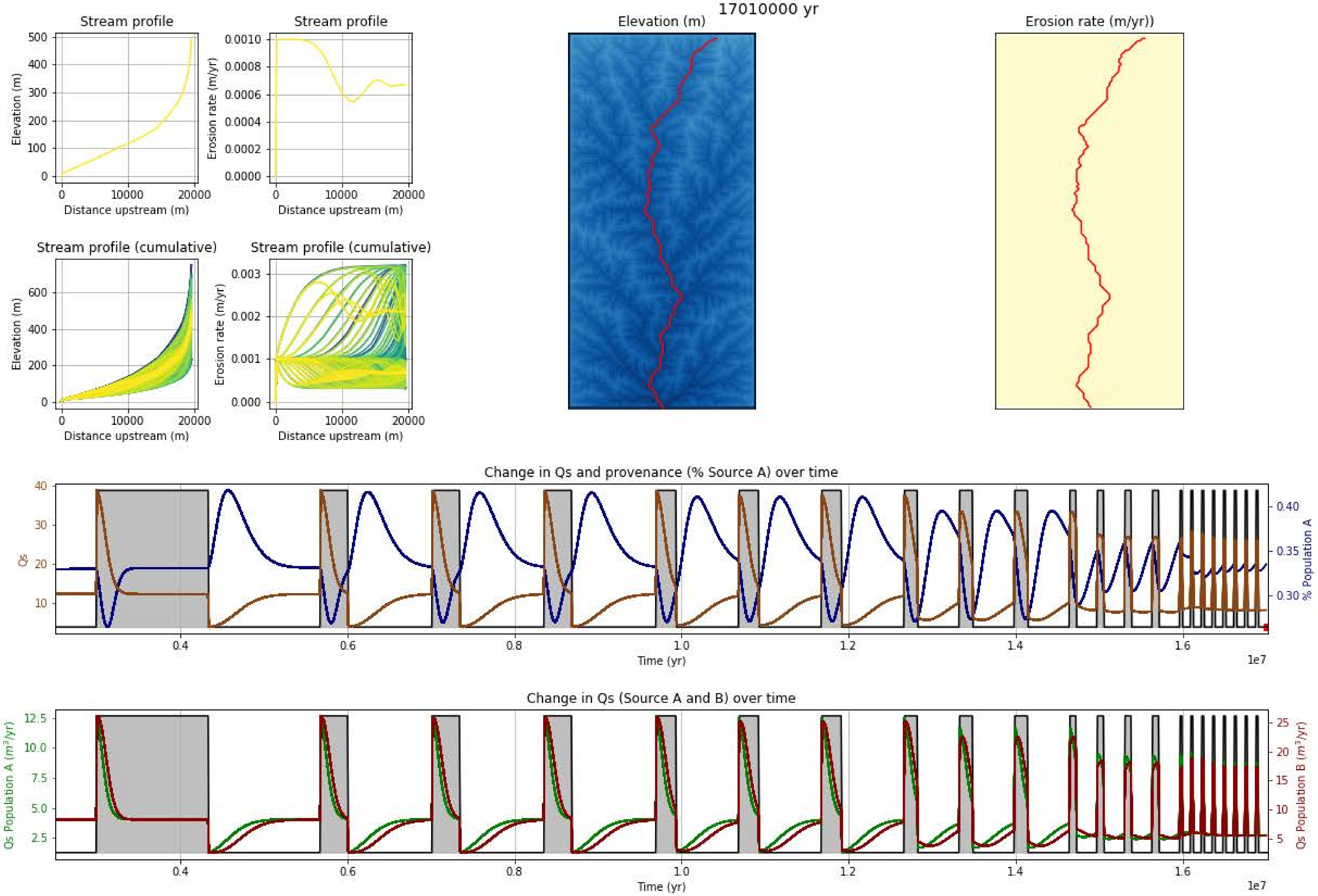

Scenario2.13

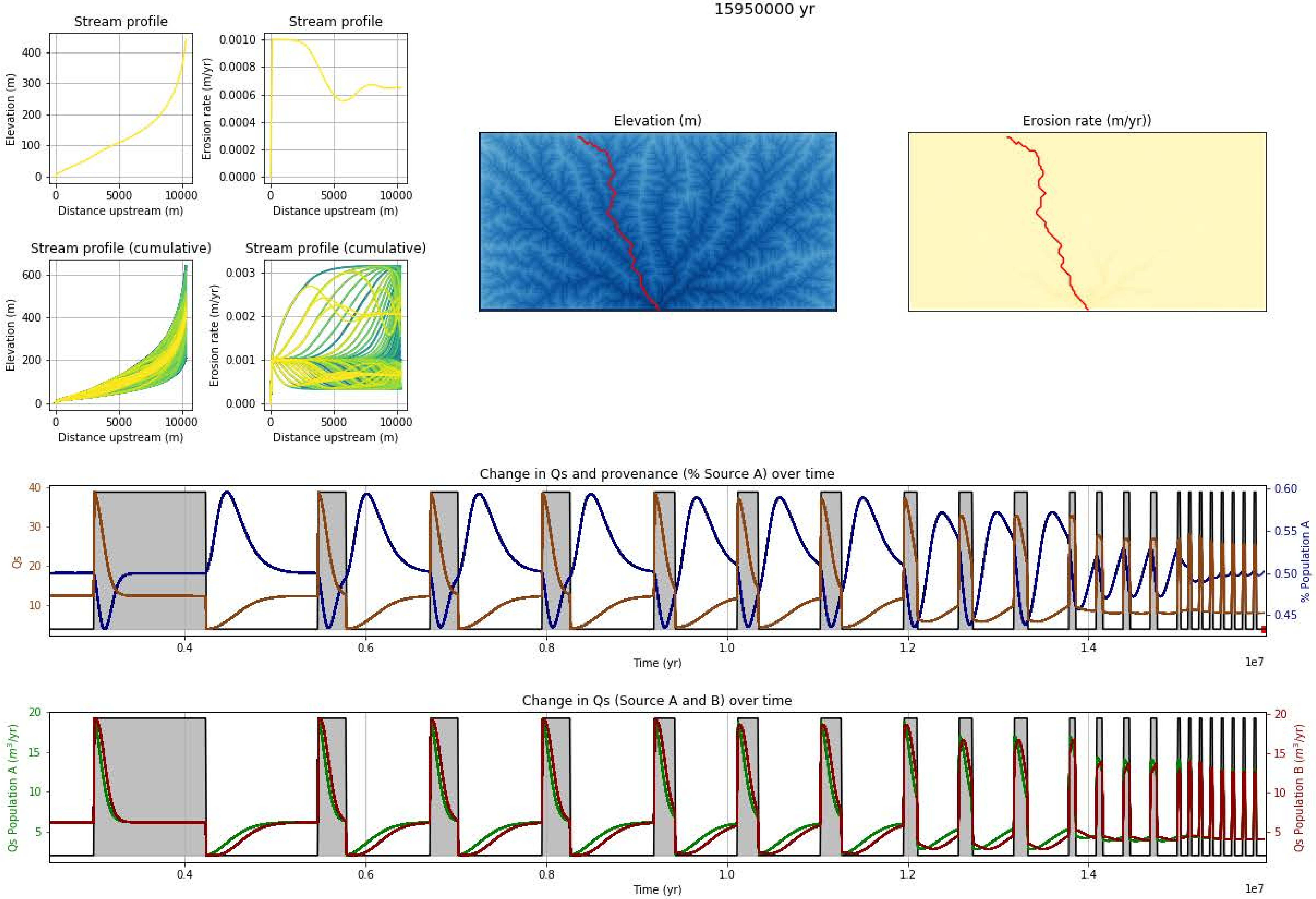

Scenario2.14

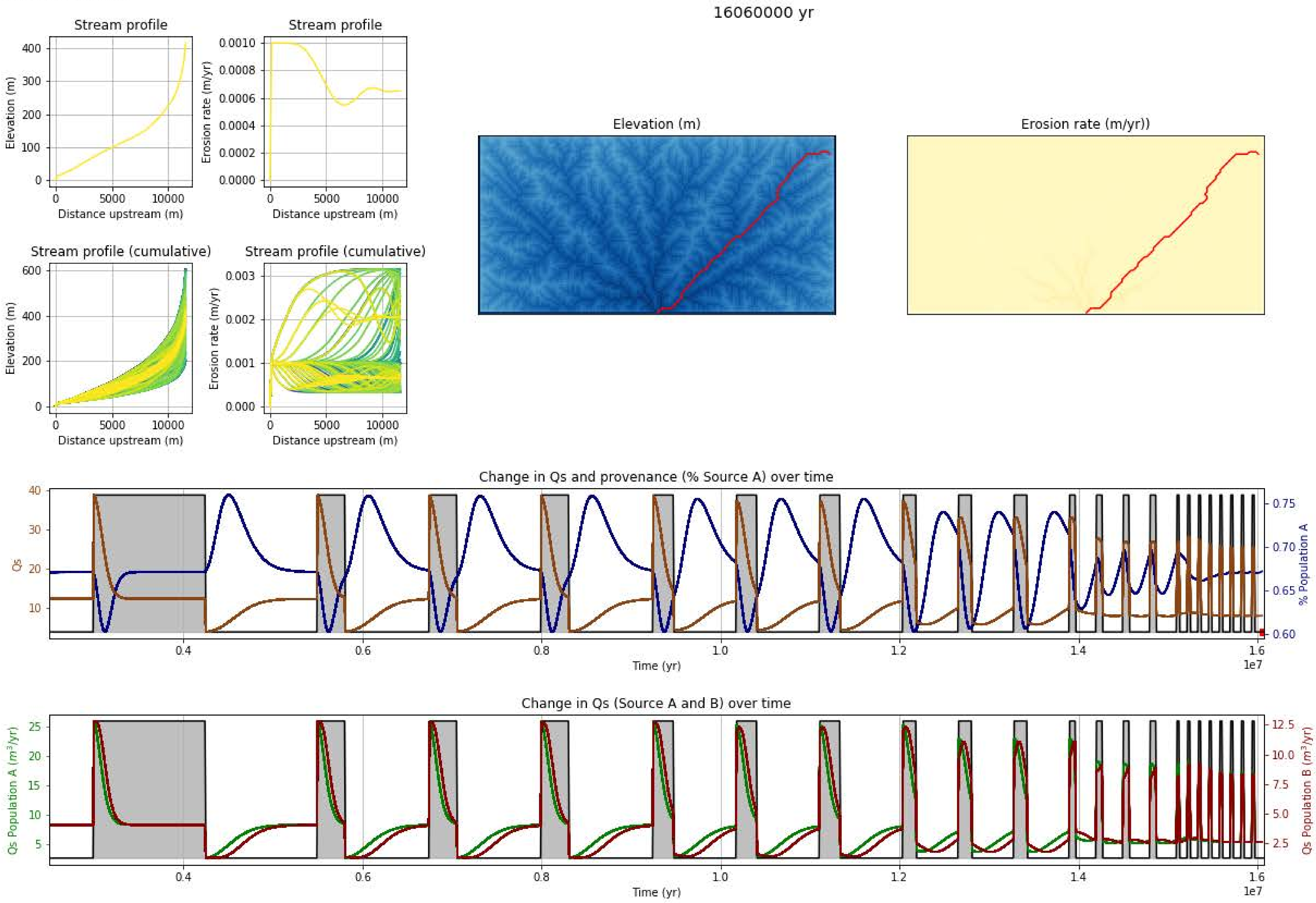

Scenario2.15

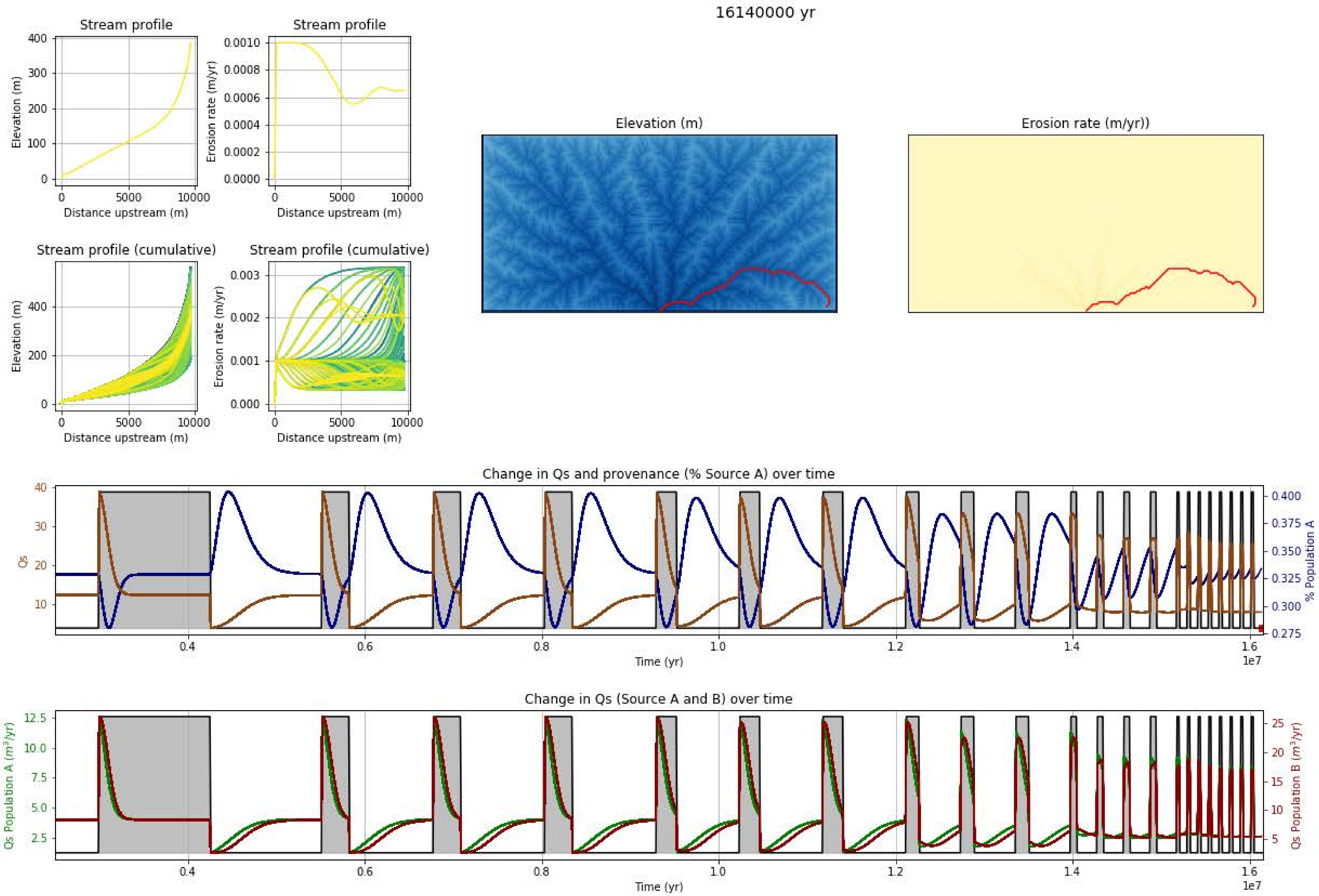

Supplement: Supplementary file 1 — Supplementary materials [file 41598_2019_39754_MOESM1_ESM.pdf]
